# Environmental changes define ecological limits to species richness and reveal the mode of macroevolutionary competition

**DOI:** 10.1111/ele.12626

**Published:** 2016-06-09

**Authors:** Thomas H. G. Ezard, Andy Purvis

**Affiliations:** ^1^ Ocean and Earth SciencesNational Oceanography Centre SouthamptonUniversity of Southampton Waterfront CampusSouthamptonSO14 3ZHUK; ^2^ Centre for Biological SciencesUniversity of SouthamptonLife Sciences Building 85Highfield CampusSouthamptonSO17 1BJUK; ^3^ Department of Life SciencesNatural History MuseumCromwell RoadLondonSW7 5BDUK; ^4^ Department of Life SciencesSilwood Park CampusImperial College LondonAscotBerkshireSL5 7PYUK

**Keywords:** Beverton–Holt, contest competition, diversification, diversity‐dependence, ecological limits, microfossil, Ricker, scramble competition

## Abstract

Co‐dependent geological and climatic changes obscure how species interact in deep time. The interplay between these environmental factors makes it hard to discern whether ecological competition exerts an upper limit on species richness. Here, using the exceptional fossil record of Cenozoic Era macroperforate planktonic foraminifera, we assess the evidence for alternative modes of macroevolutionary competition. Our models support an environmentally dependent macroevolutionary form of contest competition that yields finite upper bounds on species richness. Models of biotic competition assuming unchanging environmental conditions were overwhelmingly rejected. In the best‐supported model, temperature affects the per‐lineage diversification rate, while both temperature and an environmental driver of sediment accumulation defines the upper limit. The support for contest competition implies that incumbency constrains species richness by restricting niche availability, and that the number of macroevolutionary niches varies as a function of environmental changes.

## Introduction

The foremost regulator of life on Earth is life itself. The foundational question of whether biotic regulation restricts species richness to a finite upper limit remains nonetheless controversial (Harmon & Harrison 2015; Rabosky & Hurlbert 2015; Marshall & Quental 2016). Investigations of the existence, or not, of an upper bound to biodiversity through deep time typically focus on geological, or biological or climatic explanations (Jablonski 2008). Biological approaches often ignore geological biases and invoke the role of interspecific competition in setting diversity‐dependent limits (Sepkoski 1978; Rabosky & Hurlbert 2015), underpinned by declining speciation and/or increasing extinction rates with increasing standing diversity (Alroy 1996; Ezard *et al*. 2011). Geological approaches focus on accommodating the vagaries of the fossil record in taxonomic richness estimates by the use of sampling‐standardised curves (Alroy *et al*. 2001; Alroy 2010), residuals from models relating fossil sampling intensity to taxon counts (Smith & McGowan 2007; Lloyd *et al*. 2012) or capture‐mark‐recapture methodology (Liow & Finarelli 2014; Liow *et al*. 2015). Such approaches are motivated by the covariance between the abundance of sampled fossil material and recorded levels of biodiversity (Raup 1972; Alroy *et al*. 2001; Alroy 2010). The co‐dependence of biological, geological and climatic change extends to the covariation between the origination of geological sediments and of biological lineages (Peters 2005). This mutual dependence compromises the extrapolation of intraspecific ecological competition and equilibrial population ecology to a macroevolutionary setting (Harmon & Harrison 2015).

The traditional diversity‐dependent patterns are a negative relationship between standing taxonomic richness and diversification rate or speciation rate, or a positive relationship between richness and extinction rate (Alroy 1996; Wiens 2011; Cornell 2013). These traditional diversity‐dependent signatures evoke macroevolutionary competition, but do not reveal how biotic interactions generated them (Jablonski 2008) nor whether a finite upper bound constrains species richness (Marshall & Quental 2016). Alternative modes of macroevolutionary competition are increasingly being postulated (Cornell 2013; Voje *et al*. 2015). This expansion of macroevolutionary modes echoes an analogous proliferation through population ecology (Brännström & Sumpter 2005) and the polarisation into either ‘scramble’ or ‘contest’ competition (Hassell 1975).

**Figure 1:**
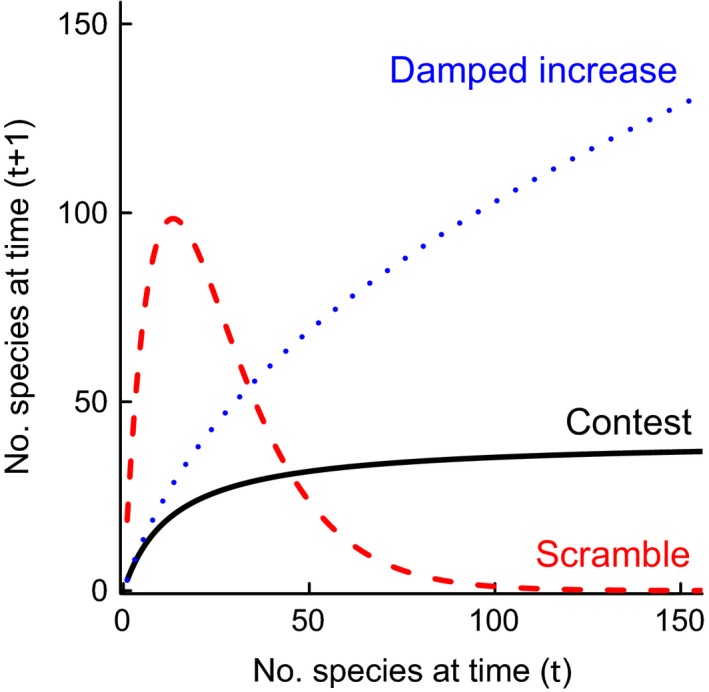
Schematic of contest (black solid line), scramble (red dashed line) and damped increase (blue dotted line) dynamics. Two dynamical features indicate scramble rather than contest competition: more rapid growth at low diversity and abrupt extinction pulses of negative, rather than zero, net change at high diversity (forms of contest competition are always non‐decreasing). Parameters (see Table 1): *r *= *k*_1_ = 3, *K *=* *40, *k*_2_ = *r/K *=* *0.075 and *c *=* *0.5. *r* is the per‐lineage diversification rate, *K* the finite upper ecological limit and *c* the competition coefficient.

Under compensatory contest competition, a constant number of successful individuals get the precise amount of resource they require, which is a fixed quantity (Varley *et al*. 1973). Scramble competition is overcompensatory: the limiting resource is shared equally among all competing individuals (Hassell 1975). At low densities, each scrambling individual therefore receives a large portion of resource (more than it needs for survival and reproduction) and population growth is very high. As abundance rises, there eventually comes a particular density when resource sharing is equivalent to contest competition (Fig. 1). Beyond this point, ‘the exactly equal partitioning of the resource sharing…[causes] an abrupt change from complete survival to complete mortality’ (Hassell 1975; see also Fig. 1).

Macroevolutionary analogues of intraspecific population ecology pivot on the assumption of niche availability (Cornell 2013). Classical niche partitioning arguments invoke contest competition, which is a discrete‐time analogue of logistic growth where better‐adapted species exclude less well‐adapted ones (Walker & Valentine 1984). Each species receives a substantial incumbency advantage, which would be reinforced if Allee effects rapidly drive the small populations of incipient species to extinction. A macroevolutionary analogue of contest competition would arise if newly available niches could only be filled by species already in very similar niches (Grafen 1989) because most then‐extant species would be unable to occupy the new niche. Contest competition provides a mechanism for a fixed niche breadth per species. Analogous to scramble competition in population ecology, Voje *et al*. (2015) describe an ‘expansion and crash’ model of macroevolutionary competition based on models of food web construction, in which evolution towards ever‐increasing specialisation is interrupted by extinction cascades provoked by the invasion of a superior mutant into an arena of vulnerable species in vulnerable niches (Takahashi *et al*. 2013). If overall biomass is held constant, then any speciation event forces a contraction in the niche breadth of all species to accommodate the new form. Such uniform contractions to the vulnerable niches eventually make the niches of all species unviable. Scramble competition thus provides a conceptually simple mechanism for both rapid diversity overshoots and crashes.

Maintaining the tractability of direct competition for resources when moving from small‐scale experiments to vast temporal and/or spatial scales is challenging because the mode of competition is partially obscured by environmental change through time (Alroy *et al*. 2001; Marshall & Quental 2016). Climate, most often temperature as a proxy for environmental energy, has been proposed as a key potential regulator of standing diversity (Mayhew *et al*. 2012) and diversification rate (Allen *et al*. 2006; Hurlbert & Stegen 2014). Climate is nonetheless a complex, multifaceted system. Changes to the elemental composition of ocean environments, for example, will, all else being equal, change the marine community composition and the number of deep sea packages deposited in the fossil record on the seabed (Moore *et al*. 1978). Under this assumption, an increase in productivity in the open ocean would provoke an increase in both biological diversification and geological sedimentation rates. Provided that the co‐dependence between geological, biological and climatic change can be incorporated fairly, the mode of macroevolutionary competition can then be revealed by building a hierarchy of mathematical models and comparing among them statistically.

Here, we use the fossil phylogeny of 210 evolutionary species of Cenozoic Era macroperforate planktonic foraminifera (Aze *et al*. 2011) to statistically assess the evidence for alternative modes of macroevolutionary competition. The species‐level fossil record of this monophyletic clade, which exhibits the traditional diversity‐dependent pattern of decreasing diversification rates with increasing standing diversity (Fig. S4 in Ezard *et al*. 2011), is at least as complete as the most complete genus‐level macrovertebrate fossil record (Ezard *et al*. 2011). This diversity dependence in this clade could be generated by either a bounded or unbounded diversification model, however (Cornell 2013; Marshall & Quental 2016). For example, Cornell's (2013) unbounded ‘damped increase’ model represents the hypothesis that diversity begets diversity (Erwin 2008) and that, as standing diversity increases, biotic competition can slow – but not halt – diversification.

Here, we compare bounded contest and scramble competition models with the unbounded damped increase alternative and abiotic ‘null’ models (Table 1). We address two principal questions. First, which mode of macroevolutionary competition best describes the dynamics of species richness? Second, is the dominant mode consistent between a fixed environment and a dynamic scenario in which environmental changes define the diversification rate and upper ecological limit?

## Methods

### The data

#### Macroperforate planktonic foraminifera

**Figure 2:**
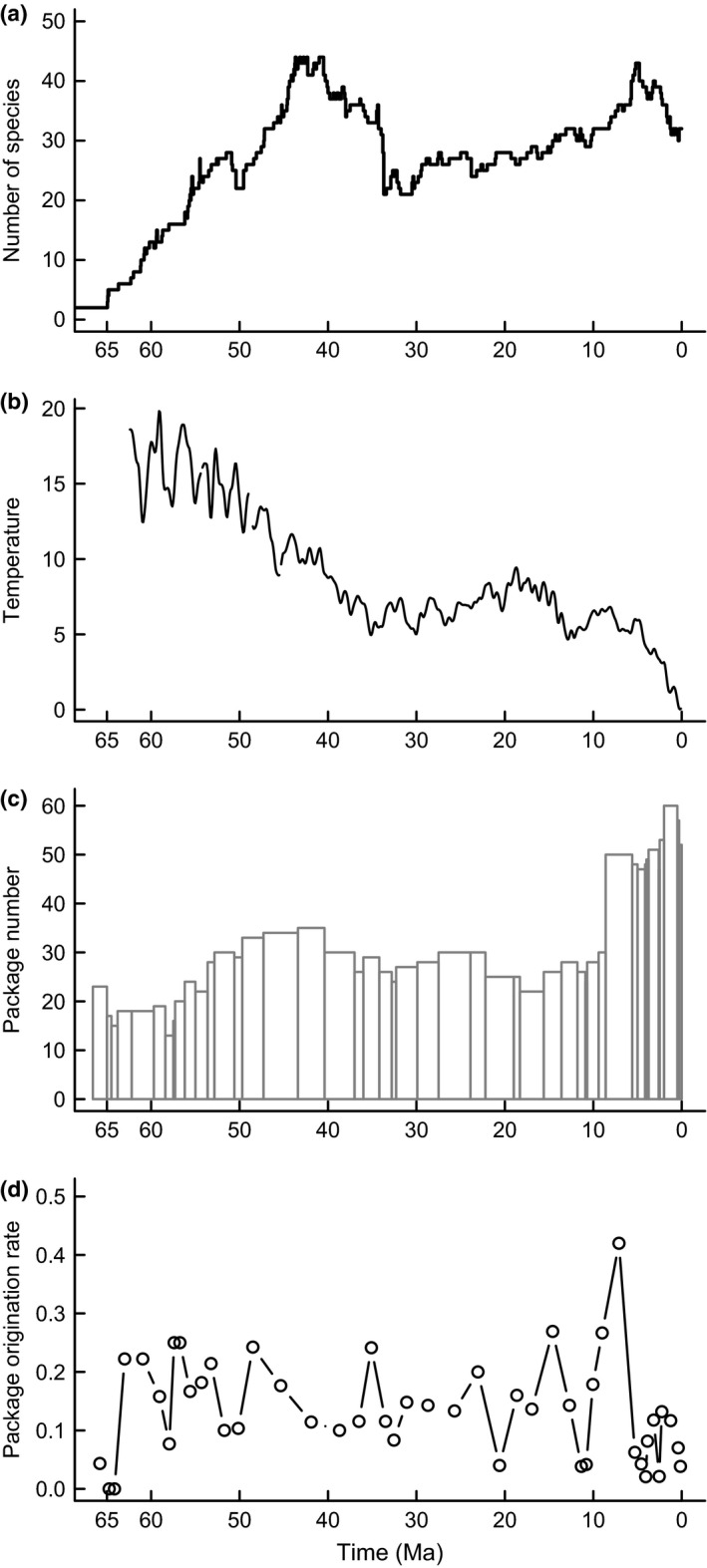
The raw data: (a) the number of evolutionary species of macroperforate planktonic foraminifera (Aze *et al*. 2011); (b) the deep sea temperature reconstruction from Mg/Ca isotopes compiled by Cramer *et al*. (2011); the (c) number of packages (Peters *et al*. 2013) and (d) the rate of package origination per geological zone (Peters *et al*. 2013).

Species‐level phylogenies of Cenozoic Era macroperforate planktonic foraminifera were presented by Aze *et al*. (2011) and contain palaeontologically calibrated ages for each speciation and extinction event within this monophyletic clade. The phylogenies were constructed using a typical microfossil, stratigraphic approach: a more‐or‐less literal reading of the fossil record to assign specimens to species‐level taxa identified from morphology. This approach is meaningful because of the abundance of this group's fossil record: these species have on average at least an 81% chance of being detected per million‐year interval throughout their existence (Ezard *et al*. 2011). Aze *et al*. (2011) aimed to eliminate the artificial ‘speciation’ and ‘extinction’ events that arise when anagenetic morphological change leads to the naming of a new form without cladogenetic lineage splitting (Pearson 1998), inferring 210 biological lineages on a phylogeny of evolutionary species (Simpson 1951). These evolutionary species are defined by breaks in the continuity of morphospace occupation between lineages, rather than the first expression of a suite of characters as in the traditional morphospecies concept (Pearson 1998). Evolutionary species counts were used for all analyses (Fig. 2a).

#### Environmental dynamics

Although climate change is complex and multifaceted, temperature is the ubiquitous single variable assumed to drive biotic responses (Mayhew *et al*. 2012; Hurlbert & Stegen 2014). We used the mean‐centred Cramer *et al*. (2011) deep ocean temperature reconstruction for the last 62.4 Myr (Fig. 2b), parameterised from Mg/Ca isotope record of deep sea carbonates using the conversion equation of Lear *et al*. (2010), as a proxy for changing conditions in the surface ocean inhabited by the focal planktonic clade. The often‐used $\delta^{18}O$ proxy (Zachos *et al*. 2001) conflates temperature, ice sheet volume and depth stratification changes, unlike Mg/Ca.

Macrostratigraphy is a dynamic quantitative approach based on the temporal dynamics of gap‐bounded rock packages (Peters 2005). The total number of packages (Fig. 2c) and rate at which new rock packages form (Fig. 2d) are key macrostratigraphic quantities describing geological dynamics. Although the species and climate data are on a global scale, we followed Peters *et al*. (2013) in restricting the macrostratigraphic data to the Atlantic because only that basin has representative sampling across its whole area and age‐structure while still capturing pole‐to‐pole changes in ocean circulation. Packages are defined as siliceous and carbonate material; successive packages are delineated by hiatuses of clay‐rich sediment. We define a bin's package origination as the number of packages that originate within it, whether or not they persist to the next bin.

#### Model fitting and selection

The species richness counts, package and climate data were amalgamated into discrete bins for analysis. All analyses were repeated for the sequence of bin lengths between 0.5 and 2 My at intervals of 100 000 years because there is no obviously correct bin length to discretise the continuous processes of sedimentation, climate change and diversification. Each discretisation leaves time series of equally spaced values. The starting point for each discretisation was varied so that all series ended at 0 Ma.

Finding no evidence to the contrary (Fig. S2), we assumed a linear relationship between temperature and diversification rate, and between package origination rate and diversification rate. In each instance, we maintained a background ‘biotic diversification’ term and included an additional abiotic component (Table 1). Species richness saturated towards higher package abundance and also appeared nonlinear to temperature change (Fig. S2), hence we assumed a flexible saturating curve of the form *aN* ^*b*^ (Table 1). For the rock package data, this form is the same as often used to model species–area relationships. All combinations of environmental regulation of per‐lineage diversification rate and upper ecological limit were considered, with the fixed parameters supplemented by abiotic terms to construct a hierarchy of increasingly complex models (Table 1).

**Table 1:** Functional forms used to model the diversity dependence of *x*_*t* + 1_, the number of species present at time *t *+* *1

Model	Scramble (Ricker 1954)	Contest (Beverton & Holt 1957)	Damped Increase (Hassell 1975)
Interpretation	*r* as diversification rate; *K* as upper limit	Discrete‐time analogue of continuous logistic growth: *k*_1_ is the diversification rate and *K* =* k*_1_/*k*_2_ is the upper limit	As contest, except the competition coefficient *c*. *c *>* *1 implies scramble competition; 0 < *c *<* *1, as here, implies damped increase competition
Fixed	$x_{t+1}\;=\;x_{t}\exp\left(r\left(1-\frac{x_{t}}{K}\right)\right)$	$x_{t+1}\;=\;\frac{k_{1}x_{t}}{\left(1+k_{2}x_{t}\right)}$	$x_{t+1}\;=\;\frac{k_{1}x_{t}}{{\left(1+k_{2}x_{t}\right)}^{c}}$
Dynamic diversification	$x_{t+1}\;=\;x_{t}\exp\left(\left(r+wT\right)\left(1-\frac{x_{t}}{K}\right)\right)$	$x_{t+1}\;=\;\frac{x_{t}\left(k_{1}+aT\right)}{\left(1+k_{2}x_{t}\right)}$	$x_{t+1}\;=\;\frac{x_{t}\left(k_{1}+wT\right)}{{\left(1+k_{2}x_{t}\right)}^{c}}$
Dynamic upper limit	$x_{t+1}\;=\;x_{t}\exp\left(r\left(1-\frac{x_{t}}{aP^{b}}\right)\right)$	$x_{t+1}\;=\;\frac{k_{1}x_{t}}{\left(1+\left(aP^{b}\right)x_{t}\right)}$	$x_{t+1}\;=\;\frac{k_{1}x_{t}}{{\left(1+\left(aP^{b}\right)x_{t}\right)}^{c}}$
Dynamic diversification & upper limit	$x_{t+1}\;=\;x_{t}\exp\left(\left(r+wT\right)\left(1-\frac{x_{t}}{aP^{b}}\right)\right)$	$x_{t+1}\;=\;\frac{x_{t}\left(k_{1}+\alpha T\right)}{\left(1+\left(aP^{b}\right)x_{t}\right)}$	$x_{t+1}\;=\;\frac{x_{t}\left(k_{1}+wT\right)}{{\left(1+\left(aP^{b}\right)x_{t}\right)}^{c}}$

The fixed models of competition imply that any change in species richness is an invariant consequence of biotic interactions according to the appropriate functional form. While the no competition (abiotic) models include diversity in the previous bin, they do not encode that diversity in a model of biotic competition. The dynamic models used the temperature reconstruction of Cramer *et al*. (2011) using the Lear *et al*. (2010) parameterisation of deep sea carbonates as a climate proxy and number (upper limit) or origination (diversification rate) of carbonate and siliceous sediment packages as geological macrostratigraphic proxies (Peters *et al*. 2013). The models of no competition took the same form as the dynamic replacements, i.e. *x*_*t*+1_ = *x*_*t*_(*r *+ *wT*) for dynamic origination rate where *w* is the abiotic diversification component driven by mean‐centred temperature *T* with *r* the background biotic rate. Analogously, *P* is the number of rock packages and related to *K* through a species‐area relationship *x*_*t*+1_ = *x*_*t*_(*aP* ^*b*^). Note that *x*_*t*+1_ refers to the more recent year and that both parameters determine the finite upper limit in contest competition. Although the table only contains certain combinations of environmentally driven diversification rate and upper limits, the global model set was assessed (Supporting Information).

Each additional level of complexity is represented by an additional parameter, which makes our model hierarchy amenable to model selection in the same way as multiple regression. We used the Akaike Information Criterion corrected for small sample size (AICc, Burnham & Anderson 2002) to assess the level of statistical support for each model in the global set. Model‐averaged results were obtained by multiplying each model's predictions by its Akaike weight, and then summing these weighted predictions across all models within a given bin. The Akaike weight quantifies the probability that a given model is the correct one of those being compared. Model‐ and time‐averaged results were obtained treating each bin equally.

All models were fitted in the R environment (R Core Team 2015) using the nlsLM function in the minpack.LM library. nlsLM is a modification of the standard nls function (Pinheiro & Bates 2000) and uses the Levenberg–Marquardt algorithm to provide more robust searching of parameter space from the starting estimates. As with nls, model fitting is by least squares and by regressing species richness at the end of the bin against a function of species richness at the start of the bin (Table 1). The approach can which can be equivalent methodologically to phenomenological approaches based on ratio or residual diversification rates (population growth rates in Coulson *et al*. 2008), but avoids biased inference when explanatory variables are correlated (Freckleton 2002). The supplementary information contains code and data to run these analyses for the 1 MY bin size. All data used here have previously been published elsewhere (Zachos *et al*. 2001; Aze *et al*. 2011; Cramer *et al*. 2011; Peters *et al*. 2013).

## Results

**Figure 3:**
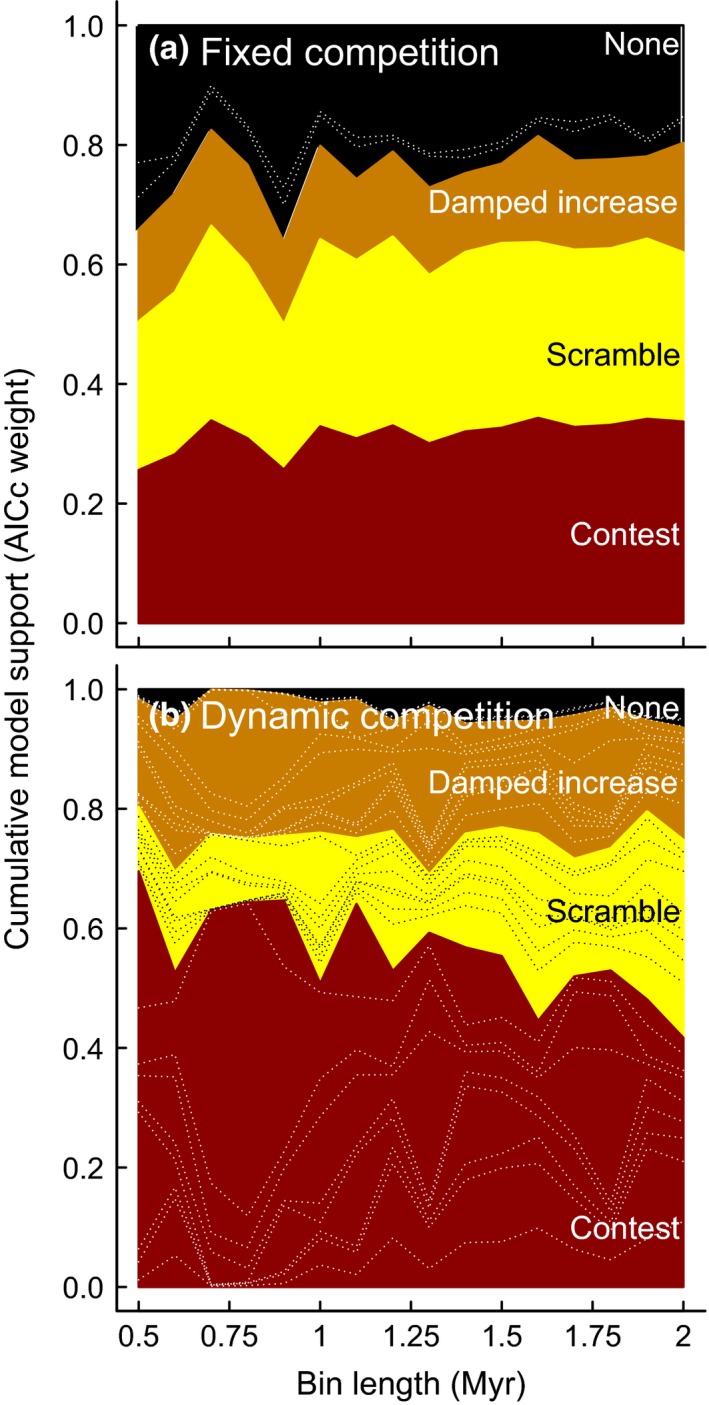
Akaike (AICc) weights indicate a signature of biotic competition assuming constant (a) and dynamic (b) functional forms (Table 1). While support for scramble is slightly greater than contest competition assuming fixed parameters, the reverse is true once the parameters vary with environmental change. Akaike weights can be interpreted as the probability that a given model is correct given those being compared. Dashed lines indicate support for particular models. See Supporting Information Table S1 for AICc scores, which, unlike Akaike weights, vary systematically with bin size (Fig S1).

Assuming an unchanging per‐lineage rate of diversification rate and fixed upper ecological limit to species richness through the entire Cenozoic Era, the three biotic models of macroevolutionary competition received the sum of 76% mean support across all bin lengths, with the four abiotic models sharing the remainder (Fig. 3a and Table S1). Under this fixed scenario, the mean support for a finite upper limit to species richness was 61%, split equally between contest (29.2%) and scramble competition (31.5%). Unbounded competition had mean support of 15%: the median competition coefficient across all bin lengths was 0.19 with an upper 95% confidence limit of 0.65. The exclusion of 1 from this confidence interval indicates that the damped increase model represents a distinct unbounded alternative to the bounded contest and scramble models, which would have been implied by a competition coefficient equal to 1, or greater than 1, respectively. Although these models represent either bounded or unbounded scenarios, all assume unchanging parameters of biotic regulation (Table 1) throughout the Cenozoic Era. This is a strong assumption if we hypothesise that changing environmental resources affect the outcome of competition (Alroy 1996; Foote 2010; Marshall & Quental 2016).

Environmental regulators of biotic competition were incorporated in our models by replacing the fixed per‐lineage diversification rate and upper ecological limit with dynamic analogues allowing these parameters to vary systematically with geological and temperature change (Table 1). Under this environmentally dependent scenario, mean support for bounded competition rose from 61 to 75% as mean support for the four abiotic models dropped from 24 to 3% (Fig. 3). Unlike the fixed case, the dynamic models strongly favour contest over scramble competition, particularly in the shortest bin lengths where the logistic‐type contest competition had around six times more support than the expansion‐and‐crash scramble alternative (Fig. 3; Tables S1–S4). In dynamic contest and damped increase competition, both geological and temperature changes alter the upper ecological limit (contest) or diversification slowdown (damped increase) and can therefore generate geologically rapid rises and falls in diversity as niche availability tracks the changing environment.

**Figure 4:**
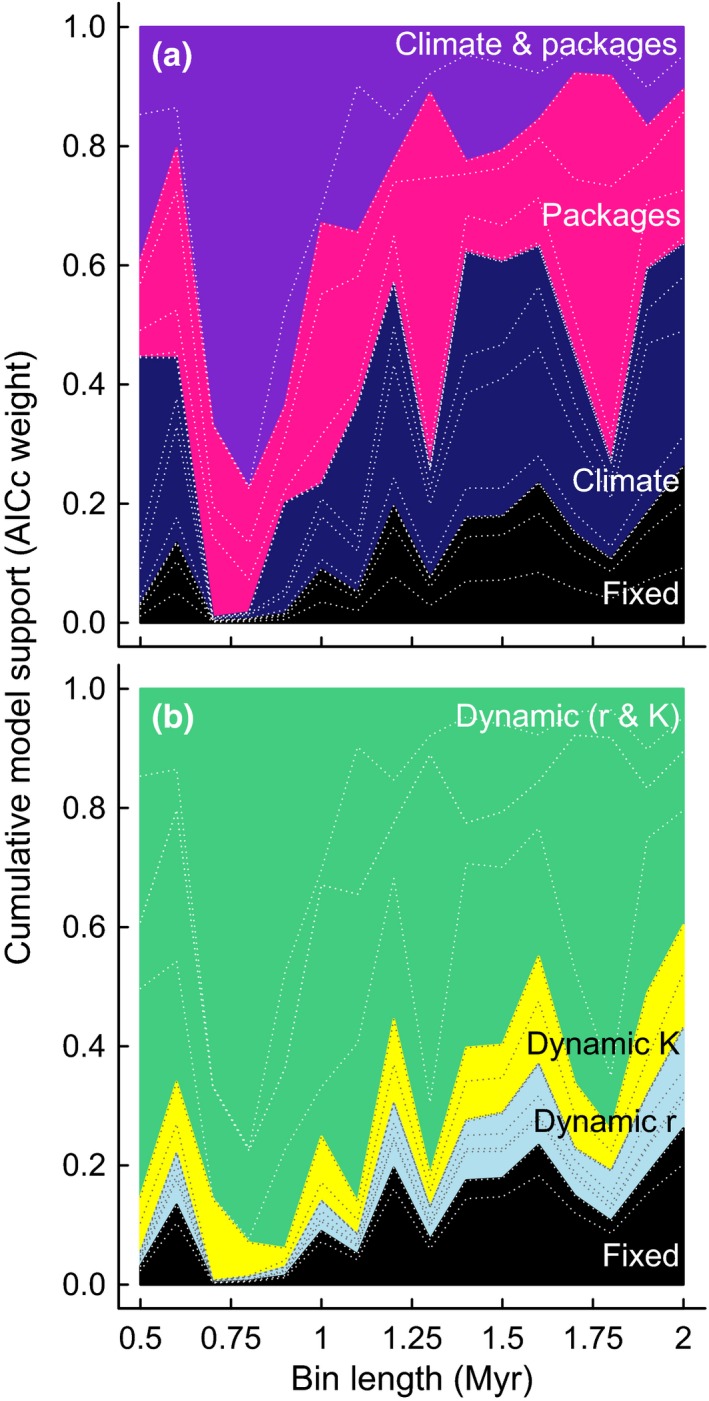
Akaike (AICc) weights for model combinations grouped by (a) whether geological and/or climatic change leaves a signature in the diversity dynamics or (b) whether diversification rate *r* and/or upper ecological limit *K* responds to climatic and/or geological change. Gaps between dashed lines give the support for particular models within the grouping – the model class with most support (package‐related upper ecological limit and temperature‐related diversification rate) is above the highest dashed line in both panels. Akaike weights can be interpreted as the probability that a given model is correct given those being compared. See Tables S1–S4 for AICc scores, which, unlike Akaike weights, vary systematically with bin size (Fig. S1).

The power to detect if temperature and geological changes determine an upper limit and/or per‐lineage diversification rate depends on the temporal resolution of analysis. In shorter bins, the evidence for the interdependent roles of temperature and geological change is strong (Fig. 4), and contest competition clearly outperforms scramble (Fig. 3b). In particular, the single combination with majority support in any bin size features temperature‐driven diversification rate and a temperature‐ and geology‐regulated upper ecological limit to species numbers (Fig. 4a). In this sweet spot of temporal resolution, the interdependent roles of environmental changes in shaping the biotic response are clear.

**Figure 5:**
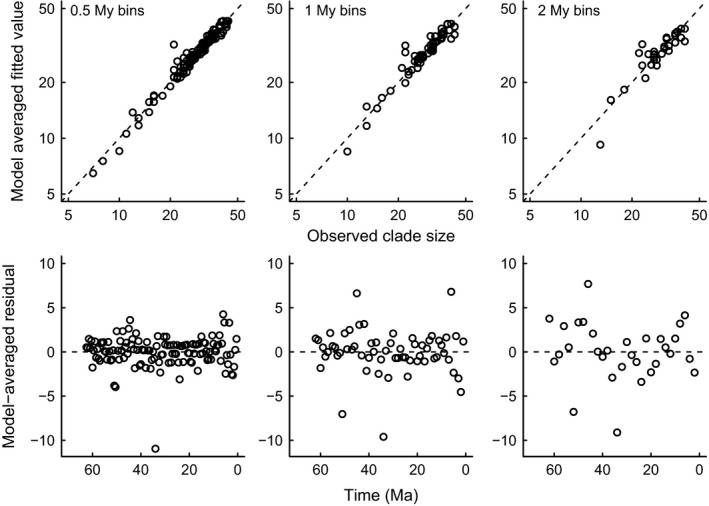
Model‐averaged predictions explain at least 80% of the observed variation in species richness from one time bin to the next (top row; the dashed line is *y* = *x*, i.e. a perfect fit) and the residuals do not indicate a temporal pattern to any error (bottom row; the dashed line is a residual of 0, i.e. a perfect fit). There is no evidence of autocorrelation in the model‐averaged residuals (Fig. S7). As bin size decreases, the variance explained by the model‐averaged predictions increases to 95%, which reiterates the importance of high‐resolution analysis to unpick co‐dependent geological, biological and climatic dynamics.

Bin lengths must, in general, be short enough to avoid sudden changes being diluted beyond detectability: models fitted to longer bins have similar Akaike weights, implying little power to identify the dominant mode of competition (Fig. 3) or how geological and climatic change regulate it (Fig. 4). Long time bins also shorten the length of time series, which reduces statistical power and amalgamates heterogeneous conditions into the same interval. This separation of environmental cause and effect means that models fitted to shorter bins explain more of the observed variation: the squared correlation between observed and model‐averaged fitted values increased from 80% in 2 MY bins, through 89% in 1 MY bins up to 95% in 0.5 MY bins (Fig. 5).

## Discussion

Despite the intuitive appeal of a finite limit on a finite Earth, whether macroevolutionary competition can generate an upper bound to species richness remains controversial (Harmon & Harrison 2015; Rabosky & Hurlbert 2015; Marshall & Quental 2016). For evidence to be compelling, Harmon & Harrison (2015) demanded species‐level studies assessing statistical support among alternative modes of biotic competition. Although verbal interpretations of dynamic upper ecological limits exist (Alroy 1996; Foote 2010; Harmon & Harrison 2015; Marshall & Quental 2016), this is the first statistical evidence comparing alternative forms of macroevolutionary competition regulated by geological and temperature change with all drivers on a level playing field.

We show overwhelming evidence that species richness in this clade is regulated by biotic competition (97%, Fig. 3b), the strength of which varies through time as a function of environmental change (Fig. 4). The environment‐driven parameters have positive coefficients, indicating that higher temperatures and higher sedimentation increase diversification rates and, where appropriate, any upper ecological limit. Biotic competition probably (75%) generates an environmentally determined upper limit to species richness, and the filling of niche space as the clade expands towards its bound likely occurs by the assumptions of logistic growth (Sepkoski 1978; Marshall & Quental 2016). Our models conceptualise intraclade competition for a limiting environmental resource, inspired by the seminal phytoplankton evidence for ecological competition (Tilman 1981). We therefore consider intraspecific competition within this monophyletic clade, rather than seeing if competition among particular types of species drives the waxing and waning of dominant ecologies (Ezard *et al*. 2011; Silvestro *et al*. 2015).

The support for package‐related change (Fig. 4a) suggests a key role of the geological record, but could in principle reflect either a literal sampling bias or some common environmental factor that generates both a biological and geological response (Alroy *et al*. 2001; Peters 2005). Given the ‘essentially complete’ (Marshall & Quental 2016) fossil record of this clade, the sampling bias interpretation is much less likely than the common environmental factor. If sampling bias were the dominant signal, we should expect a strong positive correlation between per‐species detection probability and observed diversity. We do not see this signal: Spearman rank correlations between first differences in these variables were close to zero (0.097, −0.055 and −0.114 for 0.5, 1 and 2 MY bins respectively) and not statistically significant (Table S5). A change from 2 to 0.5 MY bins only decreases the percentage of species that has a complete fossil record at that temporal resolution from 81 to 74% (Ezard *et al*. 2011). Peters *et al*. (2013) required only 7 of their 73 Atlantic sites to detect all of the modern morphospecies they analysed. Taken together, these numbers suggest sampling adequacy, which is more important than sampling completeness (Lloyd *et al*. 2012). We conclude therefore that a common environmental driver probably explains the improved fit of models including the sediment package dependency.

An enticing common driver is productivity. Energy‐rich environments support larger populations and provide more ways to construct a species‐specific niche (Hurlbert & Stegen 2014). Carbonate availability and higher rates of siliceous sedimentation may indicate higher productivity in the surface ocean (Moore *et al*. 1978), but changes in local sedimentation rates could also be driven by altered ocean circulation and/or dissolution in the water column. In some locations, higher surface productivity will lead to greater deposition of sediment on the ocean floor, but it is improbable that such a direct link applies consistently across the whole Atlantic basin. The package data we use correlate positively with other productivity proxies used elsewhere (Fig. S5, e.g. Steeman *et al*. 2009), but these alternatives are similarly difficult to interpret (Supporting Information). Although beyond the scope of this study, disentangling how sediment packages relates to primary productivity in the surface ocean would be possible using a spatially explicit analysis across distinct depth gradients. Such an analysis would also strengthen the evidence for ecological limits by assessing whether saturated communities at their upper ecological limits occur throughout the spatial ranges shared by co‐occurring species (Rabosky & Hurlbert 2015).

The fossil record provides direct information on past diversity, but interpretation is nearly always hindered by restricted temporal (Gingerich 2003) and taxonomic resolution (Benton 1997; Jablonski 2008). Even a fossil record as complete as that of Cenozoic Era macroperforate planktonic foraminifera returns a lot of very similar AICc scores among these models of macroevolutionary competition (Tables S1–S4), but restricting the model comparison to the best‐performing variants yields very similar results to Fig. 3 (Fig. S6). Ideally, all analyses of diversity dependence would take a detailed lower level approach by analysing ecology‐specific extinction and speciation probabilities to generate diversification rates (e.g. Ezard *et al*. 2011; Silvestro *et al*. 2015) because there are many routes to statistically equivalent time series of species richness (Coulson *et al*. 2008) and because changing climatic conditions impact speciation probability and extinction risk differentially (Ezard *et al*. 2011).

The three classes of models investigated here (Table 1) allow us to investigate alternative modes of macroevolutionary competition among species (Cornell 2013; Voje *et al*. 2015) rather than resorting to the traditional correlations between speciation, extinction and diversification rates with standing diversity (Alroy 1996). Each of the three models assessed can be derived from lower level interactions (Brännström & Sumpter 2005), which provides the opportunity to model how interactions among agents (individuals or populations, for example) scale up to the emergent phenomenon of species richness trajectories. Acknowledging that species will not directly interact across all their range and that local communities are often invasable (Harmon & Harrison 2015), here we prefer to use the fossil phylogeny with its consistently applied species concepts rather than extract species networks or biomass estimates from online databases. Such drilling down might elucidate more ways in which macroecological interactions generate macroevolutionary dynamics, but higher level patterns need not correspond in any simple way to lower level processes: the marine invertebrates, for example, show a single equilibrium without the component clades doing so (Alroy 2010).

Our macroevolutionary analogues of population ecology models invoke niche saturation and incumbency advantages as generating mechanisms, but do not test it explicitly. To do so would be valuable and move beyond the restrictive assumption that counts of species richness adequately represent ecological roles in communities. In reality, estimates of biomass, which Tilman (1981) controlled, and the frequency of functional traits would be more educated metrics to study ecosystem functioning (Mace *et al*. 2014). The dominance of contest over scramble competition, particularly in shorter bin lengths (Fig. 3), leads to the hypothesis that niche‐defining traits of most species will evolve from closely related species and/or those in similar existing niches, with rarer jumps in species richness potentially associated with evolutionary innovations such as the hosting of photosynthetic algal symbionts (Ezard *et al*. 2011).

The challenge of disentangling the correlated geological, climatic and biological signals has meant that, until recently, it has been hard to know whether large‐scale changes in species diversity through time reflected poor sampling (Raup 1972), poor taxonomic resolution (Benton 1997) or diversity‐dependence (Sepkoski 1978). Our models show how a sufficiently complete and fine‐grained fossil record strongly supports a more dynamic diversity‐dependence than is usually considered (Figs 3 and 4).

## Authorship

THGE and AP designed research and wrote the paper; THGE analysed the data.
