# Supplementary material for: Environmental changes define ecological limits to species richness and reveal the mode of macroevolutionary competition
Source: Ecol Lett. 2016 Jun 9;19(8):899–906. doi: 10.1111/ele.12626 (PMC4999050; doi:10.1111/ele.12626)
Supplement: Supplementary file 1 [file ELE-19-899-s001.pdf]

Supplementary Information: Environmental changes define ecological limits to species richness and reveal the mode of macroevolutionary competition

Thomas H. G. Ezard [a,b,1] and Andy Purvis[c,d,2]
[a] Ocean and Earth Sciences, National Oceanography Centre Southampton,
University of Southampton Waterfront Campus, Southampton, SO14 3ZH, UK.
[b] Centre for Biological Sciences, University of Southampton,
Life Sciences Building 85, Highfield Campus, Southampton SO17 1BJ
[c] Department of Life Sciences, Natural History Museum, London.
[d] Department of Life Sciences, Silwood Park Campus, Imperial College London.
[1] Correspondence: t.ezard@soton.ac.uk;
Tel.: +44 (0)23 8059 2985; Fax: +44 (0)23 8059 3059.
[2] andy.purvis@nhm.ac.uk

CONTENTS

Introduction **2**

Amalgamating and discretising the data **4**

Diversity (Aze *et al.*, 2011) 4

Temperature: the Mg/Ca compilation of Cramer *et al.* (2011) 4

Geological variation: the Atlantic package compilation of Peters *et al.* (2013) 5

Final data structure 7

Functional forms of biotic competition **7**

Fitting the fixed biotic models 7

Comparing the models using AICc (Fig. S1) 8

Akaike weights and the dependence of AICc scores on bin length 9

Adding geological and climatic complexity (Fig. S2) 9

A note on AIC comparisons and parameter numbers 10

Abiotic models without competition 12

Fitting models of geological and climate driven diversity dependence 12

Scramble competition (Ricker models) 12

Contest competition (Beverton-Holt models) 15

Damped increase competition (Hassell models) 16

Results **19**

Model-averaged predictions (Fig. S3) 19

AICc scores (Tables S1-S4) 19

Alternative productivity proxy metrics (Figs. S4 & S5) 19

Linear regressions of rock package number against species richness (Table S5) 27

No qualitative difference in model support between the global set and best performing models (Fig. S6) 27

No evidence of auto-correlation in model-averaged residuals (Fig. S7) 29

Bibliography **29**

INTRODUCTION

All analyses were performed in the freely distributed R environment (R Core Team, 2015). This supplement uses the `knitr` (Xie, 2012), `paleoPhylo` (Ezard & Purvis, 2009), `mgcv` (Wood, 2006), `nlme` (Pinheiro & Bates, 2000) `paleoTS` (Hunt, 2006), `xtable` and `minpack.lm` packages:

```
# set global chunk options
opts_chunk$set(fig.path='figure/minimal-', fig.align='center', fig.show='hold',
```

```
  tidy=FALSE, fig.width=5, fig.height=5, cache=FALSE, par=TRUE)
pdf.options(encoding="CP1251")
options(replace.assign=TRUE,width=80)
```

```
Sys.time()

   [1] "2016-07-12 09:26:14 BST"

sessionInfo()

   R version 3.3.0 (2016-05-03)
   Platform: x86_64-apple-darwin13.4.0 (64-bit)
   Running under: OS X 10.10.5 (Yosemite)

   locale:
   [1] en_GB.UTF-8/en_GB.UTF-8/en_GB.UTF-8/C/en_GB.UTF-8/en_GB.UTF-8

   attached base packages:
   [1] stats     graphics  grDevices utils     datasets  methods   base

   other attached packages:
   [1] paleoTS_0.5-1      mgcv_1.8-12        paleoPhylo_1.0-108 ape_3.4
   [5] xtable_1.8-2       knitr_1.13         nlme_3.1-127       minpack.lm_1.2-0

   loaded via a namespace (and not attached):
    [1] codetools_0.2-14 lattice_0.20-33  foreach_1.4.3    grid_3.3.0
    [5] formatR_1.4      magrittr_1.5     evaluate_0.9     highr_0.6
    [9] stringi_1.1.1    Matrix_1.2-6     iterators_1.0.8  tools_3.3.0
   [13] stringr_1.0.0    mnormt_1.5-4
```

The aim of this supplement is to facilitate recreation of these results. It is not exhaustive: the sections of R code contain the script to run an analysis for a single bin length (1 My) rather than looping through all bin sizes as in the manuscript. Supplementary figures referenced from the main text are are interspersed at appropriate points. Please direct any further queries or requests (including for the `.Rnw` file that is passed to `knitr`) to `t.ezard@soton.ac.uk`.

All of the data has been previously published elsewhere. You can source those files under the various distribution licences, although we provide our compilation of 1My bins to facilitate reproduction. The climate data is published as appendices to Cramer *et al.* (2011); the phylogenetic relationships of Cenozoic Era macroperforate planktonic foraminifera as online appendices to Aze *et al.* (2011); and the package data in Table S1 of Peters *et al.* (2013).

```
## Warning in file(file, "rt"):  cannot open file '/Users/te1e12/Dropbox/mechanistic diversity
dependence/jgrc12191-sup-0015-ts05.txt':  No such file or directory

## Error in file(file, "rt"):  cannot open the connection

## Warning in file(file, "rt"):  cannot open file '/Users/te1e12/Dropbox/mechanistic diversity
dependence/peters.csv':  No such file or directory

## Error in file(file, "rt"):  cannot open the connection
```

AMALGAMATING AND DISCRETISING THE DATA

The first step is to generate a discretised version of the data sources.

Diversity (Aze *et al.*, 2011)

Diversity counts were obtained using the `rwb` function in `paleoPhylo`. `aL` is the evolutionary species phylogeny available as an appendix to Aze *et al.* (2011). The diversity curve is given in Fig. 2A.

```
bl <- 1 #bin length of 1 MY
aze <- rwb(aL, bl, st=67)
#str(aze)
yt <- aze$N
lt <- length(yt)
#* append the extant data to this
disc <- data.frame(dte=aze$binStart, xt=yt, yt=c(yt[2:lt],sum(aL$en==0)))
disc <- rbind(disc, c(0, 32, 32))

#add column of natural logarithm of the species richness
disc$lnn <- log(disc$yt/disc$xt)

#curtail to the range in the Cramer data
disc <- disc[disc$dte<=62.5,]
lt <- length(disc$yt)
```

Temperature: the Mg/Ca compilation of Cramer *et al.* (2011)

A generalised additive model was applied to the Magnesium/Calcium compilation of Cramer *et al.* (2011) parameterised using Lear *et al.* (2010). First, amalgamate the climate data into 1 My bins.

```
for(i in 1:(lt-1))
  {
  nwTime <- disc$dte[i]- bl# + min(c(bl, .45))
  b4Time <- disc$dte[i]                  #i.e. calculate mean climate in the bin
  whr1 <- which(round(cramer11$Age,1)==round(max(c(0.1,nwTime)),1))
  whr2 <- which(round(cramer11$Age,1)==round(min(c(62.4,b4Time)),1))
  tmp <- whr1:whr2

  bintemps <- na.omit(cramer11$Temperature[tmp])
  if(length(bintemps)>0)
    {
    #mean-centered
    disc$c11[i]  <- mean(bintemps)-mean(cramer11$Temperature, na.rm=TRUE)
    }
  }

  Error in which(round(cramer11$Age, 1) == round(max(c(0.1, nwTime)), 1)):  object 'cramer11'
not found
```

The `c11` column contains mean-centered mean temperature reconstructions per bin.

Geological variation: the Atlantic package compilation of Peters *et al.* (2013)

Define columns `totpack` and `orgpack` as the total number of packages and total number of originating packages per epoch, respectively. Note that for `orgpack` we use packages that both originate within the time interval and persist into the next (more recent) interval (Peters *et al.*, 2013) as well as packages that originate and terminate within the time interval because the resolution of the package data is often lower than the discretised diversity counts.

```
peters$totpack <- rowSums(peters[,3:6])

  Error in is.data.frame(x):  object 'peters' not found

peters$orgpack <-  (peters$X.FL + peters$X.Ft)/peters$totpack

  Error in eval(expr, envir, enclos):  object 'peters' not found

#peters$orgpack <-  (peters$X.Ft)/peters$totpack
#not huge differences if both or a single is used, but using both
# gives more continuous results curves
#use both because the interval for consistency should be the bin,
# which is finer than the epoch
```

Then, calculate the mean number of packages (`pack`) per million years and the rate of package

origination (opack) within each time bin (Fig. 2C, D).

```
disc$pack <- disc$opack <- NA
for(j in 1:(lt-1))
{
dt1 <- disc$dte[j]
dt2 <- dt1-bl                          #packages in bin
st <- which(peters$age_bottom>=dt1)[1]
en <- which(peters$age_bottom>dt2)[1]
if(st==en)
  {
  disc$pack[j] <- peters$totpack[st]
  disc$opack[j] <- peters$orgpack[st]
  }
if(st!=en)
  {
  if((st-en)==1)
    {
    pk1 <- (dt1 - peters$age_bottom[st:en][-1])*peters$totpack[st]
    pk2 <- abs((dt2 - peters$age_bottom[st:en][-1])*peters$totpack[en])
    disc$pack[j] <- pk1 + pk2
    pk1 <- (dt1 - peters$age_bottom[st:en][-1])*peters$orgpack[st]
    pk2 <- abs((dt2 - peters$age_bottom[st:en][-1])*peters$orgpack[en])
    disc$opack[j] <- pk1 + pk2
    }
  if((st-en)>=2)
    {
    if(j==62) {agebtm <- c(0, peters$age_bottom[en:st])}
    if(j!=62) {agebtm <- peters$age_bottom[(en-1):st]}
    #agebtm
    ageinbin <- (agebtm)
    ageinbin[1] <- dt2
    ageinbin[length(ageinbin)] <- dt1
    #ageinbin

    prp <- diff(ageinbin)#(diff(ageinbin)/diff(agebtm))*diff(ageinbin)
    disc$pack[j] <- sum(peters$totpack[st:en]*prp)
    disc$opack[j] <- sum(peters$orgpack[st:en]*prp)
    }
  }
}
```

```
Error in which(peters$age_bottom >= dt1):  object 'peters' not found
```

FINAL DATA STRUCTURE

`disc` now contains all the relevant information to run the models. The final line (included for housekeeping purposes only) can be removed and the first 6 lines inspected:

```
disc <- disc[-c(lt),]
head(disc)

     dte xt yt         lnn opack pack
   6  62  7 10 0.35667494    NA   NA
   7  61 10 13 0.26236426    NA   NA
   8  60 13 13 0.00000000    NA   NA
   9  59 13 15 0.14310084    NA   NA
   10 58 15 16 0.06453852    NA   NA
   11 57 16 18 0.11778304    NA   NA
```

The columns of interest are `xt` (diversity at time t); `yt` (diversity at time $t+1$, x_{t+1} in Table 1); `c11` (the temperature reconstruction in the focal bin); `pack` (the number of packages in the focal bin); `opack` (the origination rate of packages in the focal bin). The final column is the empirical diversity dependence `lnn` as calculated in the first code chunk of this subsection.

This is the raw material for fitting the different models of biotic competition and available in the online supplement.

FUNCTIONAL FORMS OF BIOTIC COMPETITION

FITTING THE FIXED BIOTIC MODELS

The advantage of the `nlsLM` function in `minpack.lm` over `nls` is that it is computationally more exhaustive in searching parameter space using the Levenberg-Marquardt algorithm (Watson, 1978) so it can deal with "less good" initial parameter estimates and still converge on a robust solution.

```
nlc <- nls.lm.control(maxiter = 1000, maxfev=2000)
Ricker <- nlsLM(yt ~ xt*exp(r*(1-(xt/K))), start=list(r=1.5, K=35),
    data=disc, control=nlc)
BevHlt <- nlsLM(yt ~ (k1*xt)/(1+k2*xt), start=list(k1=1, k2=0),
    data=disc, control=nlc)
Hassll <- nlsLM(yt ~ (k1*xt)/((1+k2*xt)^cc), start=c(k1=1.2, k2=0.002, cc=1),
```

```
    data=disc, control=nlc)
```

COMPARING THE MODELS USING AICC (FIG. S1)

These models therefore represent ways in which competition for a limiting resource among, in this instance, species generate different diversity dynamics. The Akaike Information Criterion (Burnham & Anderson, 2002) is a way of summarising the deviance explained by a candidate model. It attempts to find the best model within the set of candidates and is calculated:

$$AIC = 2k - 2 * (log\ likelihood)$$

where k is the number of parameters and $log\ likelihood$ is the log-likelihood of the model. It therefore provides a trade-off between variance explained and parameters used. A version with improved behaviour is the Akaike Information Criterion corrected for small sample size, AICc:

$$AICc = 2k - 2 * (log\ likelihood) + \frac{2k(k+1)}{n-k-1} = AIC + \frac{2k(k+1)}{n-k-1}$$

where n is the number of observations. As n increases, the AICc converges on the AIC. The following function calculates AICc for `nls` objects.

```
aicc <- function(mm)
  {
  #### calculates AICc for an nls object
  if(class(mm)!="nls") stop("object is not of class 'nls'")

  strmm <- summary(mm)
  K <- strmm$df[1]
  AICc <- AIC(mm) + (2*K*(K+1))/strmm$df[2]
  return(AICc)
  }
```

```
aicc(Ricker)

   [1] 300.921

aicc(BevHlt)

   [1] 300.8228

aicc(Hassll)

   [1] 302.3112
```

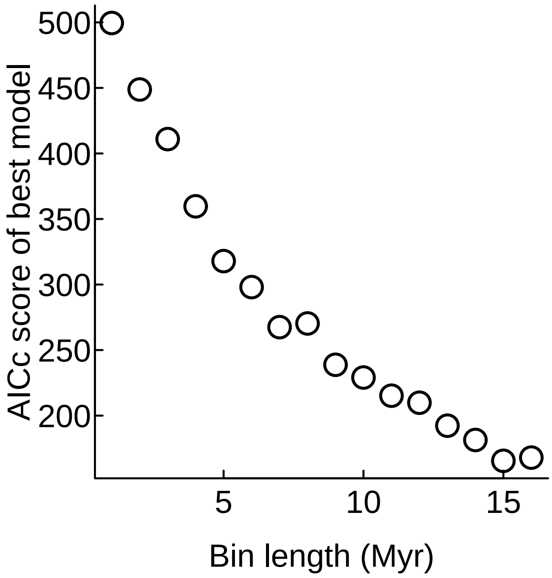

Figure S1: The similar support of Akaike weight probabilities in support of either scramble or contest competition does not vary systematically with bin length. Identical support for both for both forms of biotic competition would be indicated by a horizontal line at 0.5. All scramble and contest competition models are included in these cumulative sums. We use Akaike weights for the figure because the model likelihood varies systematically with bin length.

There is very little to choose between these models without incorporating additional complexity via the package and climate data. Therefore, for now, we restrict ourselves to the comparison of Ricker, Beverton-Holt and Hassell models as representatives of scramble, contest and flexible forms of competition. Other functional forms are available (see Brännström & Sumpter, 2005, for a review). Of the alternative forms, the theta-Ricker had strong support for some bin sizes when climate variation was also included but is not considered because of concerns about its ability to represent accurately the strength of the density dependence (Clark *et al.*, 2010) and because it cannot be derived from underlying competition among (here) species (Brännström & Sumpter, 2005).

AKAIKE WEIGHTS AND THE DEPENDENCE OF AICC SCORES ON BIN LENGTH

We report Akaike weights, calculated as $w_i = \frac{exp(-\frac{1}{2}\Delta_i(AICc))}{\sum_{k=1}^{K} exp(-\frac{1}{2}\Delta_i(AICc))}$ in the main manuscript because, unlike full AICc scores (Tables S1-S4, Fig. S1), they do not scale systematically with bin size. Different amounts of raw variation in the compiled data sets generate the systematic variation in deviance, which underpins AICc, in Fig. S1.

ADDING GEOLOGICAL AND CLIMATIC COMPLEXITY (FIG. S2)

The next step is to increase the complexity of the models using the lagged and contemporaneous climate and contemporaneous package data.

```
par(mfrow=c(2,2), bty='l', las=1, mar=c(3.5, 4, .5, .2), mgp=c(2,.25,0), tcl=.3)
#figure S2
with(disc, plot(c11, lnn,  ylab="Empirical clade growth", xlab="Temperature"))

  Error in plot(c11, lnn, ylab = "Empirical clade growth", xlab = "Temperature"):  object
'c11' not found

with(disc, plot(opack, lnn,
  xlab="Package origination rate", ylab="Empirical clade growth"))

  Warning in min(x):  no non-missing arguments to min; returning Inf

  Warning in max(x):  no non-missing arguments to max; returning -Inf

  Error in plot.window(...):  need finite 'xlim' values

with(disc, plot(c11, yt, ylab="Number of species", xlab="Temperature"))

  Error in plot(c11, yt, ylab = "Number of species", xlab = "Temperature"):  object 'c11'
not found

with(disc, plot(pack, yt, xlab="Package number", ylab="Number of species"))

  Warning in min(x):  no non-missing arguments to min; returning Inf

  Warning in min(x):  no non-missing arguments to max; returning -Inf

  Error in plot.window(...):  need finite 'xlim' values
```

A note on AIC comparisons and parameter numbers

Consider the model $w = ax + b + \epsilon$, where the epsilon are independent and identically distributed normal variables with mean 0. Compare that model to $w = ax + 5y + b + \epsilon$, where epsilon follows the same rules as before. The second model has the same number of free parameters as the first one, so all it has to do is beat the first one absolutely in fraction of variance explained (the parameter discounting in AIC is irrelevant because both models have the same number of free parameters). In the sort of mechanistic modelling we report here, the validity of the comparison, particularly how fair or unfair it is, depends on where the 5 came from.

If the 5 came from fitting the model $w = ax + cb + \epsilon$, secretly, and obtaining 5 for the best-fitting value of c, the comparison would be unfair because of the secret parameter. Alternatively, if a physical (or other strong *a priori*) law predicted the 5, then there is no need to estimate it statistically and the comparison would be justified.

Use of the palaeoclimate proxy is somewhere between these extremes. Given the phenomenological nature of the models, we resolve the issue by comparison to statistical regression models. The

Figure S2: Relationships between mean centered temperature and empirical clade growth (i.e., diversification rate), and between package number and clade growth (top) as well as between both explanatory variables and standing diversity (bottom).

competition mechanisms we seek to test emerge from comparing amongst multiple models. In statistical modelling, one has an additional coefficient to multiply the covariate by. We therefore treat the forcing function under the same logic – models with additional complexity have an additional parameter to estimate (see Tables S1-S4).

ABIOTIC MODELS WITHOUT COMPETITION

Four models without any biotic competition.

```
#### abiotic models
packageK <- nlsLM(yt ~ xt*(a*pack)^b, start=list(a=1, b=.2), data=disc, control=nlc)

  Error in nls.lm(par = start, fn = FCT, jac = jac, control = control, lower = lower, :
evaluation of fn function returns non-sensible value!

climateK <- nlsLM(yt ~ xt*(a*abs(c11))^b, start=list(a=1, b=.2),
  data=disc, control=nlc)

  Error in nlsLM(yt ~ xt * (a * abs(c11))^b, start = list(a = 1, b = 0.2), :  parameters
without starting value in 'data':  c11

climater <- nlsLM(yt ~ xt*(1+a*c11), start=list(a=1), data=disc, control=nlc)

  Error in nlsLM(yt ~ xt * (1 + a * c11), start = list(a = 1), data = disc, :  parameters
without starting value in 'data':  c11

packager <- nlsLM(yt ~ xt*(1+a*opack), start=list(a=1), data=disc, control=nlc)

  Error in nls.lm(par = start, fn = FCT, jac = jac, control = control, lower = lower, :
evaluation of fn function returns non-sensible value!
```

FITTING MODELS OF GEOLOGICAL AND CLIMATE DRIVEN DIVERSITY DEPENDENCE

Scramble competition (Ricker models)

In the models that follow, x_t is the number of species at time t, r is the diversification rate, K is the upper ecological limit ("carrying capacity" in population biology), T is the mean-centered temperature reconstruction, P the number and o_p the origination rate of macrostratigraphic packages.

The mathematical code is slightly different for scripting reasons, i.e. T is `c11` (for Cramer11, see above), o_p is `opack` and P is `pack`.

Finally, we use x_{t+1} for transparency in the manuscript and mathematical formulae, but y_t in the R data frame.

`Rick1`: Scramble competition with climate-regulated diversification rate

$x_{t+1} = x_t \exp\left((r + wT)\left(1 - \frac{x_t}{K}\right)\right)$

```
Rick1 <- nlsLM(yt ~ xt*exp((r+w*c11)*(1-(xt/K))),
  start=list(r=.1, w=0, K=20), data=disc, control=nlc)

  Error in nlsLM(yt ~ xt * exp((r + w * c11) * (1 - (xt/K))), start = list(r = 0.1, :  parameters
without starting value in 'data':  c11
```

`Rick2`: Scramble competition with climate-regulated upper ecological limit

$x_{t+1} = x_t \exp\left(r\left(1 - \frac{x_t}{(aT)^b}\right)\right)$

```
Rick2 <- nlsLM(yt ~  xt*exp(r*(1-(xt/(a*(abs(c11)^b))))),
  start=list(r=0.1, a=4, b=1.2), data=disc, control=nlc)

  Error in nlsLM(yt ~ xt * exp(r * (1 - (xt/(a * (abs(c11)^b))))), start = list(r = 0.1,
:  parameters without starting value in 'data':  c11
```

`Rick3`: Scramble competition with climate-regulated diversification rate and climate regulated upper ecological limit

$x_{t+1} = x_t \exp\left((r + wT)\left(1 - \frac{x_t}{(aT)^b}\right)\right)$

```
Rick3 <- nlsLM(yt ~ xt*exp((r+w*c11)*(1-(xt/a*(abs(c11)^b)))),
  start=list(r=0.1, w=0, a=4, b=1.2), data=disc, control=nlc)

  Error in nlsLM(yt ~ xt * exp((r + w * c11) * (1 - (xt/a * (abs(c11)^b)))), :  parameters
without starting value in 'data':  c11
```

`Rick4`: Scramble competition with package-regulated upper ecological limit

$x_{t+1} = x_t \exp\left(r\left(1 - \frac{x_t}{aP^b}\right)\right)$

```
Rick4 <- nlsLM(yt ~ xt*exp(r*(1-(xt/(a*pack^b)))),
  start=list(r=.1, a=20, b=1.2), data=disc, control=nlc)

  Error in nls.lm(par = start, fn = FCT, jac = jac, control = control, lower = lower, :
evaluation of fn function returns non-sensible value!
```

`Rick5`: Scramble competition with package origination-regulated diversification rate

$x_{t+1} = x_t \exp\left((r + wo_p)\left(1 - \frac{x_t}{K}\right)\right)$

```
Rick5 <- nlsLM(yt ~ xt*exp((r+w*opack)*(1-(xt/K))),
  start=list(r=.1, w=0, K=20), data=disc, control=nlc)

  Error in nls.lm(par = start, fn = FCT, jac = jac, control = control, lower = lower, :
evaluation of fn function returns non-sensible value!
```

`Rick6`: Scramble competition with package origination-regulated diversification rate and package-regulated upper ecological limit
$x_{t+1} = x_t \exp\left((r + wo_p)\left(1 - \frac{x_t}{aP^b}\right)\right)$

```
Rick6 <- nlsLM(yt ~ xt*exp((r+w*opack)*(1-(xt/(a*pack^b)))),
  start=list(r=.1, w=0, a=20, b=1.2), data=disc, control=nlc)

  Error in nls.lm(par = start, fn = FCT, jac = jac, control = control, lower = lower, :
evaluation of fn function returns non-sensible value!
```

`Rick7`: Scramble competition with climate-regulated diversification rate and package-regulated upper ecological limit
$x_{t+1} = x_t \exp\left((r + wT)\left(1 - \frac{x_t}{aP^b}\right)\right)$

```
Rick7 <- nlsLM(yt ~ xt*exp((r+w*c11)*(1-(xt/(a*pack^b)))),
  start=list(r=.1, b=1.2, w=0, a=20), data=disc, control=nlc)

  Error in nlsLM(yt ~ xt * exp((r + w * c11) * (1 - (xt/(a * pack^b)))), :  parameters
without starting value in 'data':  c11
```

`Rick8`: Scramble competition with package origination-regulated diversification rate and climate-regulated upper ecological limit
$x_{t+1} = x_t \exp\left((r + wo_p)\left(1 - \frac{x_t}{aT^b}\right)\right)$

```
Rick8 <- nlsLM(yt ~ xt*exp((r+w*opack)*(1-(xt/(a*(abs(c11)^b))))),
  start=list(r=.1, w=0, a=20, b=1.2), data=disc, control=nlc)

  Error in nlsLM(yt ~ xt * exp((r + w * opack) * (1 - (xt/(a * (abs(c11)^b))))), :  parameters
without starting value in 'data':  c11
```

Contest competition (Beverton-Holt models)

In the models of contest competition, the ecological upper limit, K in the Ricker model of scramble competition, is given by $\frac{k_1}{k_2}$.

BH1: Contest competition with climate-regulated diversification rate $\dfrac{x_t(k_1 + wT)}{(1 + k_2 x_t)}$

```
BH1 <- nlsLM(yt ~ (xt*(k1 + w*c11))/(1+k2*xt),
  start=list(k1=0.2, k2=.2, w=1), data=disc, control=nlc)

  Error in nlsLM(yt ~ (xt * (k1 + w * c11))/(1 + k2 * xt), start = list(k1 = 0.2, :  parameters
without starting value in 'data':  c11
```

BH2: Contest competition with climate-regulated upper ecological limit $\dfrac{x_t k_1}{(1 + (aT^b)x_t)}$

```
BH2 <- nlsLM(yt ~ (xt*k1)/(1+(a*(abs(c11)^b))*xt),
  start=list(k1=1.2, a=0, b=1.2), data=disc, control=nlc)

  Error in nlsLM(yt ~ (xt * k1)/(1 + (a * (abs(c11)^b)) * xt), start = list(k1 = 1.2, :
parameters without starting value in 'data':  c11
```

BH3: Contest competition with climate-regulated diversification rate and climate regulated upper ecological limit: $\dfrac{x_t(k_1 + wT)}{(1 + ((aT)^b)x_t)}$

```
BH3 <- nlsLM(yt ~ (xt*(k1 + w*c11))/(1+(a*(abs(c11)^b))*xt),
  start=list( k1=0, w=1, a=4, b=1.2), data=disc, control=nlc)

  Error in nlsLM(yt ~ (xt * (k1 + w * c11))/(1 + (a * (abs(c11)^b)) * xt), :  parameters
without starting value in 'data':  c11
```

BH4: Contest competition with package-regulated upper ecological limit $\dfrac{x_t k_1}{(1 + (aP^b)x_t)}$

```
BH4 <- nlsLM(yt ~ (xt*k1)/(1+(a*pack^b)*xt),
  start=list(k1=0, a=20, b=1), data=disc, control=nlc)

  Error in nls.lm(par = start, fn = FCT, jac = jac, control = control, lower = lower, :
evaluation of fn function returns non-sensible value!
```

BH5: Contest competition with package origination-regulated diversification rate $\frac{x_t(k_1 + wo_p)}{(1 + k_2 x_t)}$

```
BH5 <- nlsLM(yt ~ (xt*(k1 + w*opack))/(1+k2*xt),
  start=list(k1=0, w=1, k2=20), data=disc, control=nlc)

  Error in nls.lm(par = start, fn = FCT, jac = jac, control = control, lower = lower, :
evaluation of fn function returns non-sensible value!
```

BH6: Contest competition with package origination-regulated diversification rate and package-regulated upper ecological limit $\frac{x_t(k_1 + wo_p)}{(1 + (aP^b)x_t)}$

```
BH6 <-  nlsLM(yt ~ (xt*(k1 + w*opack))/(1+(a*pack^b)*xt),
  start=list(k1=0, w=1, a=4, b=1.2), data=disc, control=nlc)

  Error in nls.lm(par = start, fn = FCT, jac = jac, control = control, lower = lower, :
evaluation of fn function returns non-sensible value!
```

BH7: Contest competition with climate-regulated diversification rate and package-regulated upper ecological limit $\frac{x_t(k_1 + wT)}{(1 + (aP^b)x_t)}$

```
BH7 <- nlsLM(yt ~ (xt*(k1 + w*c11))/(1+(a*pack^b)*xt),
   start=list(k1=0.2, w=.2, a=20, b=1.2), data=disc, control=nlc)

  Error in nlsLM(yt ~ (xt * (k1 + w * c11))/(1 + (a * pack^b) * xt), start = list(k1 =
0.2, :  parameters without starting value in 'data':  c11
```

BH8: Contest competition with package origination-regulated diversification rate and climate-regulated upper ecological limit $\frac{x_t(k_1 + wT)}{(1 + (aT)^b x_t)}$

```
BH8 <- nlsLM(yt ~ (xt*(k1 + w*c11))/(1+(a*(abs(c11)^b))*xt),
  start=list(k1=0.2, w=.2, a=20, b=1.2), data=disc, control=nlc)

  Error in nlsLM(yt ~ (xt * (k1 + w * c11))/(1 + (a * (abs(c11)^b)) * xt), :  parameters
without starting value in 'data':  c11
```

Damped increase competition (Hassell models)

The Hassell model modifies the Beverton-Holt through a competition coefficient c. Depending on the value of c, the model generates damped increase competition if $0 < c < 1$, contest competition

if $c = 1$ (where the model reduces to the Beverton-Holt) or scramble competition if $c > 1$. See also Fig. 1. In the following R code, we use cc instead of c because the latter is an R function. Note that we constrain $c > 0$ (Hassell, 1975; Brännström & Sumpter, 2005) using the `lower` argument.

`Hass1`: Damped increase competition with climate-regulated diversification rate $\dfrac{x_t(k_1 + wT)}{(1 + k_2 x_t)^c}$

```
Hass1 <- nlsLM(yt ~ (xt*(k1 + w*c11))/((1+k2*xt)^cc),
  start=list(k1=0, k2=.2, w=1, cc=.4), data=disc, control=nlc,
  lower=c(-Inf, -Inf, -Inf, 0.01), upper=c(Inf, Inf, Inf, 10))

  Error in nlsLM(yt ~ (xt * (k1 + w * c11))/((1 + k2 * xt)^cc), start = list(k1 = 0, :
parameters without starting value in 'data':  c11
```

`Hass2`: Damped increase competition with climate-regulated upper ecological limit $\dfrac{x_t k_1}{(1 + (aT^b)x_t)^c}$

```
Hass2 <- nlsLM(yt ~ (xt*k1)/((1+(a*(abs(c11)^b))*xt)^cc),
  start=list(k1=0, a=4, b=1.2, cc=.4), data=disc, control=nlc,
  lower=c(-Inf, -Inf, -Inf, 0.01), upper=c(Inf, Inf, Inf, 10))

  Error in nlsLM(yt ~ (xt * k1)/((1 + (a * (abs(c11)^b)) * xt)^cc), start = list(k1 = 0,
:  parameters without starting value in 'data':  c11
```

`Hass3`: Damped increase competition with climate-regulated diversification rate and climate regulated upper ecological limit: $\dfrac{x_t(k_1 + wT)}{(1 + (aT^b)x_t)^c}$

```
Hass3 <- nlsLM(yt ~ (xt*(k1 + w*c11))/((1+(a*(abs(c11)^b))*xt)^cc),
  start=list( k1=0, w=0, a=18, b=1.2, cc=1), data=disc, control=nlc,
  lower=c(-Inf, -Inf, -Inf, -Inf, 0.01), upper=c(Inf, Inf, Inf, Inf, 10))

  Error in nlsLM(yt ~ (xt * (k1 + w * c11))/((1 + (a * (abs(c11)^b)) * xt)^cc), :  parameters
without starting value in 'data':  c11
```

`Hass4`: Damped increase competition with package-regulated upper ecological limit $\dfrac{x_t k_1}{(1 + (aP^b)x_t)^c}$

```
Hass4 <- nlsLM(yt ~ (xt*k1)/((1+(a*pack^b)*xt)^cc),
  start=list(k1=0, a=20, b=1, cc=.4), data=disc, control=nlc,
  lower=c(-Inf, -Inf, -Inf, 0.01), upper=c(Inf, Inf, Inf, 10))

  Error in nls.lm(par = start, fn = FCT, jac = jac, control = control, lower = lower, :
evaluation of fn function returns non-sensible value!
```

`Hass5`: Damped increase competition with package origination-regulated diversification rate $\frac{x_t(k_1 + wo_p)}{(1 + k_2 x_t)^c}$

```
Hass5 <- nlsLM(yt ~ (xt*(k1 + w*opack))/((1+k2*xt)^cc),
  start=list(k1=0, w=1, k2=20, cc=.4), data=disc, control=nlc,
  lower=c(-Inf, -Inf, -Inf, 0.01), upper=c(Inf, Inf, Inf, 10))

  Error in nls.lm(par = start, fn = FCT, jac = jac, control = control, lower = lower, :
evaluation of fn function returns non-sensible value!
```

`Hass6`: Damped increase competition with package origination-regulated diversification rate and package-regulated upper ecological limit $\frac{x_t(k_1 + wo_p)}{(1 + (aP^b)x_t)^c}$

```
Hass6 <-  nlsLM(yt ~ (xt*(k1 + w*opack))/((1+(a*pack^b)*xt)^cc),
  start=list(k1=0, w=1, a=4, b=1.2, cc=.4), data=disc, control=nlc,
  lower=c(-Inf, -Inf, -Inf, -Inf, 0.01), upper=c(Inf, Inf, Inf, Inf, 10))

  Error in nls.lm(par = start, fn = FCT, jac = jac, control = control, lower = lower, :
evaluation of fn function returns non-sensible value!
```

`Hass7`: Damped increase competition with climate-regulated diversification rate and package-regulated upper ecological limit $\frac{x_t(k_1 + wT)}{(1 + (aP^b)x_t)^c}$

```
Hass7 <- nlsLM(yt ~ (xt*(k1 + w*c11))/((1+(a*pack^b)*xt)^cc),
   start=list(k1=0.2, w=.2, a=20, b=1.2, cc=.4), data=disc, control=nlc,
  lower=c(-Inf, -Inf, -Inf, -Inf, 0.01), upper=c(Inf, Inf, Inf, Inf, 10))

  Error in nlsLM(yt ~ (xt * (k1 + w * c11))/((1 + (a * pack^b) * xt)^cc), :  parameters
without starting value in 'data':  c11
```

`Hass8`: Damped increase competition with package origination-regulated diversification rate and climate-regulated upper ecological limit $\frac{x_t(k_1 + wT)}{(1+(aT^b)x_t)^c}$

```
Hass8 <- nlsLM(yt ~ (xt*(k1 + w*c11))/((1+(a*(abs(c11)^b))*xt)^cc),
  start=list(k1=0, w=.2, a=15, b=1.2, cc=.4), data=disc, control=nlc,
  lower=c(-Inf, -Inf, -Inf, -Inf, 0.01), upper=c(Inf, Inf, Inf, Inf, 10))

  Error in nlsLM(yt ~ (xt * (k1 + w * c11))/((1 + (a * (abs(c11)^b)) * xt)^cc), :  parameters
without starting value in 'data':  c11
```

RESULTS

MODEL-AVERAGED PREDICTIONS (FIG. S3)

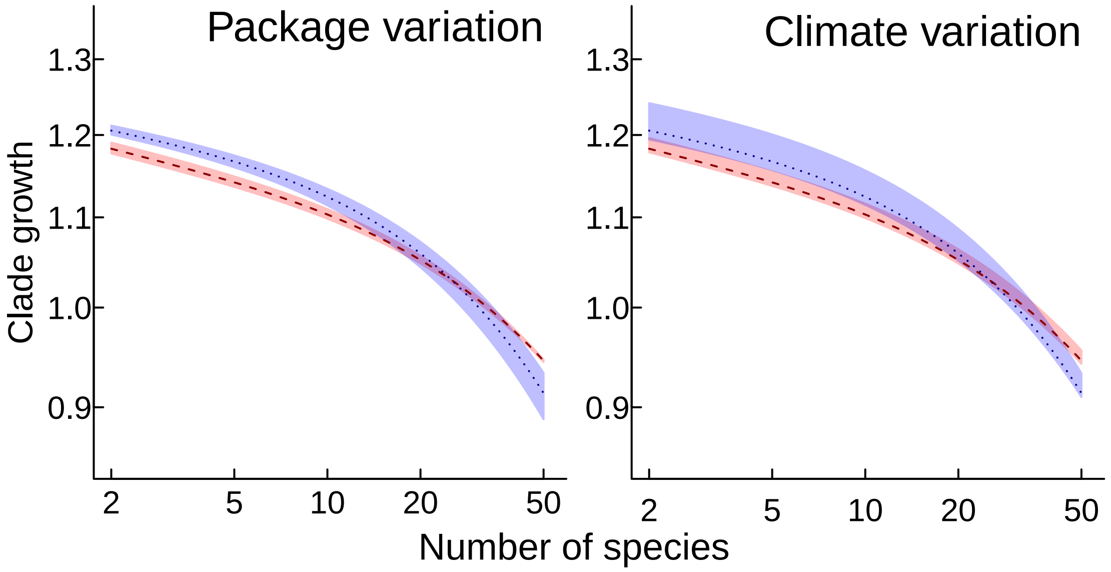

Figure S3: Model-averaged clade growth is more sensitive to climate than package variation. Coloured envelopes give predictions between the 1st and 3rd quartiles of either package (left panel) or temperature (right panel) variation while the other variable is held at its median level. Note natural logarithm scales on both axes.

AICC SCORES (TABLES S1-S4)

See overleaf.

ALTERNATIVE PRODUCTIVITY PROXY METRICS (FIGS. S4 & S5)

The appeal of a role of productivity as a potential "common cause" driver is strong, but the package data do not strongly support such an interpretation. (Peters *et al.*, 2013) defined hiatuses between

Competition type	Scramble	Contest	Damped increase	None (package K)	None (package r)	None (climate K)	None (climate r)
No. parameters	2	2	3	2	1	2	1
0.5 My bins	505.50	505.44	506.46	513.26	508.69	508.44	505.66
0.6 My bins	450.38	450.30	451.37	457.96	453.87	457.23	450.81
0.7 My bins	422.65	422.57	424.07	430.44	426.07	429.75	425.01
0.8 My bins	370.56	370.44	371.68	378.49	373.97	378.04	371.66
0.9 My bins	325.91	325.80	327.06	333.48	328.77	330.19	325.69
1 My bins	300.92	300.82	302.31	309.03	304.78	308.68	302.47
1.1 My bins	271.59	271.52	273.17	279.90	275.19	277.43	272.52
1.2 My bins	271.27	271.18	272.87	279.86	277.14	279.21	272.35
1.3 My bins	243.38	243.25	244.69	251.83	246.89	251.55	243.93
1.4 My bins	230.33	230.20	231.98	238.33	235.70	236.48	231.07
1.5 My bins	216.30	216.19	217.99	224.75	221.53	223.37	217.22
1.6 My bins	210.37	210.06	211.38	218.35	215.72	218.34	211.66
1.7 My bins	194.24	194.04	195.62	202.24	198.11	199.98	195.46
1.8 My bins	185.13	184.90	186.50	193.17	188.27	192.42	186.49
1.9 My bins	167.05	166.80	168.62	175.14	172.63	174.82	167.98
2 My bins	168.35	168.00	169.22	175.79	172.81	175.70	169.59

Table S1: Akaike Information Criterion scores corrected for small sample size, with Akaike weight (across **all** models) in brackets, for the fixed biotic and simple abiotic models that comprise Fig. 4. Akaike weights can be interpreted as the probability that a given model is correct among of those fitted. r is the per-lineage diversification rate; K is the upper limit to species richness. Compare scores along rows, i.e. within each bin size. The mean competition correlation in the damped increase (Hassell) models was 0.19, with the upper 95% bound at 0.65, i,e. well away from 1 when this form would reduce to contest competition. This difference implies that the damped increase and contest models are genuinely reflecting different modes of biotic competition here.

Code	Fixed	Rick1	Rick2	Rick3	Rick4	Rick5	Rick6	Rick7	Rick8
climate r		1		1				1	
climate K			1	1					1
package r						1	1		1
package K					1		1	1	
No. parameters	2	3	3	4	3	3	4	4	4
0.5 My bins	505.50	507.44	504.23	510.78	504.73	506.87	506.61	504.19	504.85
0.6 My bins	450.38	452.42	452.47	454.54	451.64	452.43	453.47	452.95	454.54
0.7 My bins	422.65	424.78	424.43	426.62	415.02	422.22	416.87	415.99	425.40
0.8 My bins	370.56	372.71	372.72	374.93	365.87	369.38	366.80	364.21	371.24
0.9 My bins	325.91	327.88	326.40	330.79	325.25	326.37	325.61	321.22	323.75
1 My bins	300.92	303.01	303.07	305.19	301.27	302.35	301.66	298.59	304.48
1.1 My bins	271.59	273.67	273.02	277.84	272.69	273.17	272.35	273.44	270.93
1.2 My bins	271.27	273.52	273.52	275.86	273.22	273.03	274.41	275.73	275.36
1.3 My bins	243.38	245.57	245.42	247.68	245.45	244.41	245.52	247.61	247.49
1.4 My bins	230.33	232.58	232.32	235.43	232.57	232.55	234.96	236.21	234.15
1.5 My bins	216.30	218.46	218.57	220.85	218.62	217.82	220.15	220.85	219.45
1.6 My bins	210.37	212.27	211.88	213.31	212.66	211.32	213.61	214.72	212.38
1.7 My bins	194.24	196.26	196.25	201.61	196.25	196.61	195.58	198.19	196.28
1.8 My bins	185.13	187.04	187.53	189.60	187.44	185.20	184.64	189.58	186.22
1.9 My bins	167.05	168.00	169.32	170.51	168.08	168.19	168.61	168.41	170.73
2 My bins	168.35	170.17	170.36	171.72	170.71	168.90	171.34	172.60	171.37

Table S2: Akaike Information Criterion scores corrected for small sample size, with Akaike weight (across **all** models) in brackets, for the scramble competition (Ricker) models that, with Tables S3 & S4, comprise Fig. 5. Akaike weights can be interpreted as the probability that a given model is correct among of those fitted. r is the per-lineage diversification rate; K is the upper limit to species richness. Model codes refer to section 3.6.1 in this supplement. Compare scores along rows, i.e. within each bin size.

Code	Fixed	BH1	BH2	BH3	BH4	BH5	BH6	BH7	BH8
climate r		1		1				1	
climate K			1	1					1
package r						1	1		1
package K					1		1	1	
No. parameters	2	3	3	4	3	3	4	4	4
0.5 My bins	505.44	503.61	504.27	499.56	504.69	502.86	504.48	501.34	499.56
0.6 My bins	450.30	449.11	452.40	450.52	451.56	448.83	450.99	449.25	450.52
0.7 My bins	422.57	422.49	424.38	423.34	415.25	416.35	414.40	411.00	423.34
0.8 My bins	370.44	369.76	372.61	371.63	365.95	365.16	364.32	359.74	371.63
0.9 My bins	325.80	323.91	326.43	319.99	325.15	321.44	322.70	317.92	319.99
1 My bins	300.82	300.46	302.98	302.67	301.09	298.08	299.74	298.09	302.67
1.1 My bins	271.52	270.39	273.01	267.53	272.60	268.05	270.14	268.64	267.53
1.2 My bins	271.18	270.34	273.43	272.20	273.07	272.61	274.78	270.59	272.20
1.3 My bins	243.25	241.65	245.33	243.98	245.28	238.82	241.18	242.04	243.98
1.4 My bins	230.20	229.52	232.21	229.09	232.46	231.80	234.06	231.77	229.09
1.5 My bins	216.19	215.22	218.44	215.58	218.51	217.24	219.54	217.38	215.58
1.6 My bins	210.06	209.88	211.66	212.14	212.30	212.30	214.71	211.41	212.14
1.7 My bins	194.04	193.49	196.07	194.09	196.09	192.35	193.26	195.99	201.10
1.8 My bins	184.90	184.75	187.29	186.75	187.15	181.45	183.52	186.19	186.75
1.9 My bins	166.80	165.55	169.11	168.06	167.83	169.22	170.36	167.71	168.06
2 My bins	168.00	168.19	170.06	170.78	170.37	170.01	172.44	170.81	170.78

Table S3: Akaike Information Criterion scores corrected for small sample size, with Akaike weight (across **all** models) in brackets, for the contest competition (Beverton-Holt) models that, with Tables S2 & S4, comprise Fig. 5. Akaike weights can be interpreted as the probability that a given model is correct among of those fitted. r is the per-lineage diversification rate; K is the upper limit to species richness. Model codes refer to section 3.6.2 in this supplement. Compare scores along rows, i.e. within each bin size.

Code	Fixed	Hass1	Hass2	Hass3	Hass4	Hass5	Hass6	Hass7	Hass8
climate r		1		1				1	
climate K			1	1					1
package r						1	1		1
package K					1		1	1	
No. parameters	2	3	3	4	3	3	4	4	4
0.5 My bins	505.44	505.41	508.66	501.70	506.09	503.63	505.51	503.60	538.68
0.6 My bins	450.30	450.89	453.51	452.88	452.77	449.67	451.87	451.28	452.88
0.7 My bins	422.57	424.55	426.00	425.50	417.24	417.46	416.56	412.93	425.50
0.8 My bins	370.44	371.66	373.90	373.83	368.25	366.07	366.02	361.73	373.83
0.9 My bins	325.80	325.97	328.22	322.30	326.76	322.39	323.92	320.10	322.30
1 My bins	300.82	302.53	304.59	304.87	302.58	299.40	301.14	300.32	304.87
1.1 My bins	271.52	272.73	274.95	269.31	274.37	269.57	271.78	278.50	269.31
1.2 My bins	271.18	272.66	275.21	274.54	274.76	274.26	276.49	272.93	274.97
1.3 My bins	243.25	243.89	246.99	246.36	246.78	240.00	242.47	244.29	246.36
1.4 My bins	230.20	231.91	234.13	231.56	234.37	233.66	236.07	234.30	231.56
1.5 My bins	216.19	217.65	220.33	218.16	220.43	219.07	221.44	219.98	218.16
1.6 My bins	210.06	211.82	213.38	214.23	213.73	213.73	216.28	213.53	214.23
1.7 My bins	194.04	195.75	197.74	196.70	197.82	193.85	194.31	198.43	196.70
1.8 My bins	184.90	186.99	189.02	189.43	188.85	182.84	185.05	188.53	189.43
1.9 My bins	166.80	168.11	171.19	170.84	169.66	171.23	172.35	170.29	170.84
2 My bins	168.00	170.09	171.63	172.87	171.77	171.37	173.92	172.91	172.87

Table S4: Akaike Information Criterion scores corrected for small sample size, with Akaike weight (across **all** models) in brackets, for the damped increase (Hassell) models that, with Tables S2 & S3, comprise Fig. 5. Akaike weights can be interpreted as the probability that a given model is correct among of those fitted. r is the per-lineage diversification rate; K is the upper limit to species richness. Model codes refer to section 3.6.3 in this supplement. Compare scores along rows, i.e. within each bin size.

packages as periods of time when calcareous and siliceous sediments are infrequent relative to clay-rich sediment. In the deep sea, changes in calcareous and siliceous sedimentation could be driven by dissolution in the water column, changes in ocean circulation, an increase in productivity in the surface ocean or a combination thereof (Moore Jr. *et al.*, 1978). Sedimentation change is also not independent of temperature change (Bohaty & Zachos, 2004). A consequence of this interdependence has been the use of other productivity proxies, particularly the deep sea $\delta^{13}C$ record (Zachos *et al.*, 2001, 2008), which is a composite signal of export productivity in deep sea carbonates (e.g. for applications, Steeman *et al.*, 2009; Condamine *et al.*, 2013; Lazarus *et al.*, 2014). A further option to remove the binary classification in (Peters *et al.*, 2013) by modelling the *rate* of carbonate and siliceous sedimentation, rather than simply its presence. Higher rates of siliceous sedimentation have been argued to be a rough indicator of higher export productivity of phytoplankton in the overlying water column (Lazarus *et al.*, 2014). We therefore calculated these quantitates and compared them to the package data.

Firstly, extract the $\delta^{13}C$ record. Using the same NEPTUNE compilation as for the completeness statistics in the main text, we then reduced the data to the Atlantic basin only and predicted the sedimentation rate for the single site with the most complete record using a generalised additive mixed model (Wood, 2006). The first difference of the predicted values from a non-parametric smoothed spline then give a coarse metric of sedimentation rates (Fig. S4).

```
## Warning in file(file, "rt"):  cannot open file '/Users/te1e12/Dropbox/mechanistic diversity
dependence/IncWithAnalysisNames11-11-10.txt':  No such file or directory

## Error in file(file, "rt"):  cannot open the connection
```

```r
np$keep <- paste(np$Sample.Depth, np$Hole.ID, sep="-")

  Error in paste(np$Sample.Depth, np$Hole.ID, sep = "-"):  object 'np' not found

atl <- np[np$Ocean=="Atlantic",]

  Error in eval(expr, envir, enclos):  object 'np' not found

atl <- atl[!duplicated(np$keep),]#

  Error in eval(expr, envir, enclos):  object 'atl' not found

m1 <- gamm(Sample.Depth ~ s(Sample.Age, k=20) + s(Hole.ID, bs="re"), data=atl)

  Error in is.data.frame(data):  object 'atl' not found

newdf <- data.frame(Sample.Age=seq(0,62,1), Hole.ID=rep("41_366A", 63))
pp <- diff(predict.gam(m1$gam, newdata=newdf))

  Error in predict.gam(m1$gam, newdata = newdf):  object 'm1' not found

#diff means that row 1 is change from 0 to 1, i.e. lagged over previous bin
```

```
plot(-61:0, rev(pp), type='l', xlab="Time (Ma)", ylab="sedimentation rate (m/My)",
  axes=FALSE)

  Error in rev(pp): object 'pp' not found

axis(1, c(-65, -6:0*10), labels=abs(c(-65, -6:0*10)))

  Error in axis(1, c(-65, -6:0 * 10), labels = abs(c(-65, -6:0 * 10))): plot.new has not
been called yet

axis(2, 0:3*10, las=1)

  Error in axis(2, 0:3 * 10, las = 1): plot.new has not been called yet

box(bty='l')

  Error in box(bty = "l"): plot.new has not been called yet

#figure S4
disc$sedrate <- rev(pp)

  Error in rev(pp): object 'pp' not found
```

Using the functions within the `pairs` help file, Fig. S8 shows the correlation between δ^{13}C and the number of packages (i.e., Peters *et al.*'s (2013) "common cause" metric). The correlation between the sedimentation rate inferred from NEPTUNE and either δ^{13}C or the number of packages is weaker (Fig. S5).

```
climate_proxies <-disc[,c(7,9,10)]

  Error in `[.data.frame`(disc, , c(7, 9, 10)): undefined columns selected

colnames(climate_proxies) <- c("Packages", "Carbon", "Sedimentation")

  Error in colnames(climate_proxies) <- c("Packages", "Carbon", "Sedimentation"): object
'climate_proxies' not found

pairs(climate_proxies, diag.panel=panel.hist,
  lower.panel=panel.smooth, upper.panel=panel.cor)

  Error in pairs(climate_proxies, diag.panel = panel.hist, lower.panel = panel.smooth,
: object 'climate_proxies' not found

#figure S5
```

The moderate correlation between package number and δ^{13}C suggests a possible link between these two metrics. We do not adopt δ^{13}C as a viable index of productivity because it is a signal of export productivity in the focal clade: the vital effects of different species, including the presence

or absence of symbionts (Norris, 1996), violate the key assumption of equilibrium between test and water column. Furthermore, $\delta^{13}C$ does not explain the observed hiatuses in the rock record. Disentangling the merits of these interdependent hypotheses is a considerable task and beyond the scope of the coarse models presented here, but could be achieved by repeated sampling of a depth transect to identify how, if at all, dissolution contributes to the sedimentary hiatuses.

Nonetheless, as detailed in the main manuscript, the relationships between package number and species diversity is unlikely to be a strict sampling bias in a clade with such an extraordinarily abundant fossil record. Simple linear regressions support the conclusion that a sampling bias interpretation is unlikely (Table S5).

LINEAR REGRESSIONS OF ROCK PACKAGE NUMBER AGAINST SPECIES RICHNESS (TABLE S5)

Bin size (My)	Coefficient	Standard Error	p-value	Adjusted r^2	Spearman's rank correlation
0.5	0.0011	0.0076	$p > 0.05$	-0.003	0.097
1	-0.0001	0.0031	$p > 0.05$	-0.016	-0.061
2	-0.0019	0.0044	$p > 0.05$	-0.027	-0.114

Table S5: Linear regressions, and Spearman's rank correlation coefficients, illustrate that changes in rock package number is not a statistically significant predictor of changes in macroperforate planktonic foraminifera species richness during the Cenozoic Era. These regressions and correlations use the first differences in both explanatory and response variables to remove the long-term trend in the data (Fig. 2).

NO QUALITATIVE DIFFERENCE IN MODEL SUPPORT BETWEEN THE GLOBAL SET AND BEST PERFORMING MODELS (FIG. S6)

Figure overleaf.

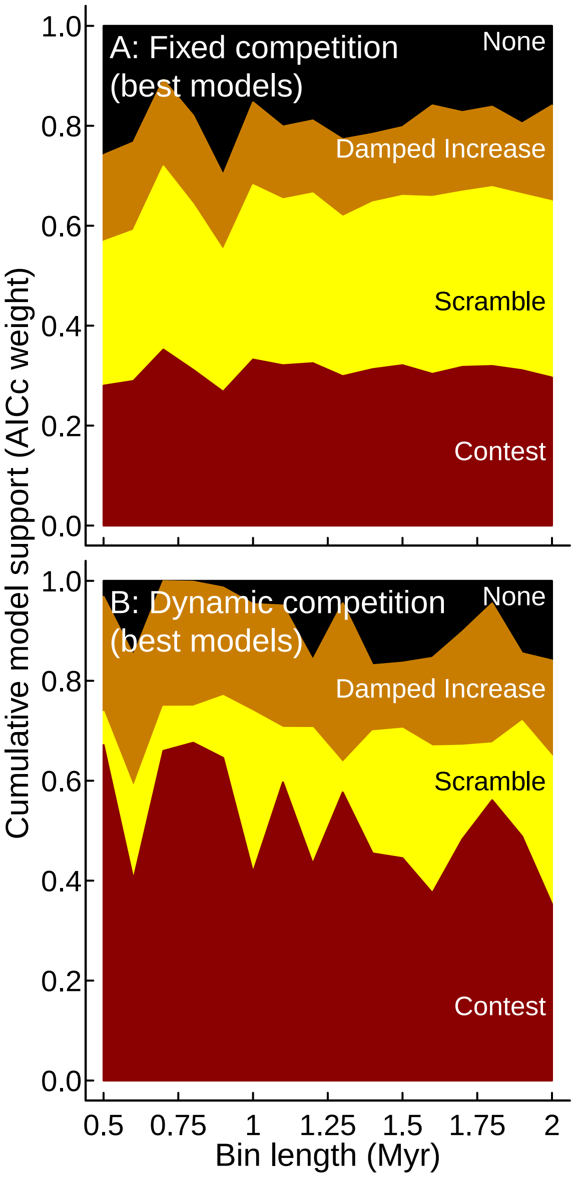

Figure S4: Akaike (AICc) weights indicate a signature of biotic competition assuming constant (A) and dynamic (B) functional forms (Table 1) using only the best performing (minimum AICc) model per category. This figure is very similar to Fig. 3 in the main manuscript, which implies our amalgamation of lots of different models into classes is not driving the patterns we see.

NO EVIDENCE OF AUTO-CORRELATION IN MODEL-AVERAGED RESIDUALS (FIG. S7)

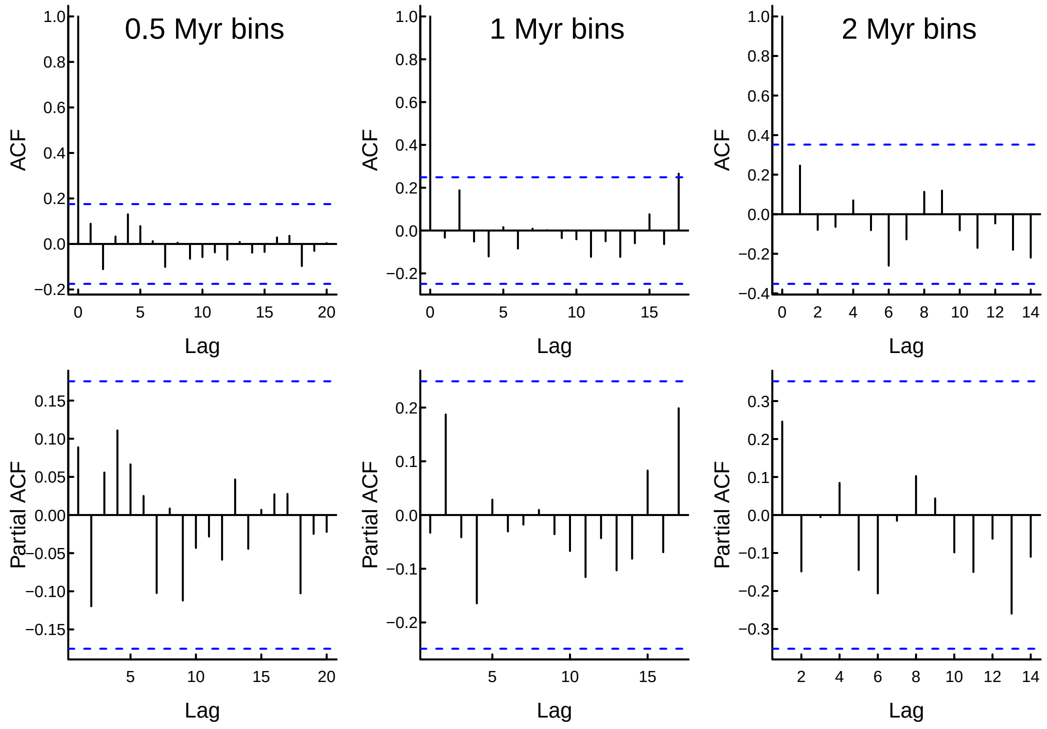

Figure S5: There is no evidence of auto-correlation in model-averaged residuals, either on the auto-correlation function (top row) or partial auto-correlation function (bottom row). Dashed lines denote a significance level of 0.05.

BIBLIOGRAPHY

Aze, T., Ezard, T. H. G., Purvis, A., Coxall, H. K., Stewart, D. R. M., Wade, B. S. & Pearson, P. N. (2011). A phylogeny of Cenozoic macroperforate planktonic foraminifera from fossil data. *Biol. Rev.*, 86, 900–927.

Bohaty, S. M. & Zachos, J. C. (2004). Significant southern ocean warming event in the late middle Eocene. *Geology*, 31, 1017–1020.

Brännström, Å. & Sumpter, D. J. T. (2005). The role of competition and clustering in population dynamics. *Proc. Roy. Soc. B*, 272, 2065–2072.

Burnham, K. P. & Anderson, D. R. (2002). *Model selection and multimodel inference. A practical information–theoretical Approach.* Springer-Verlag, New York.

Clark, F., Brook, B. W., Delean, S., Reşit Akçakaya, H. & Bradshaw, C. J. A. (2010). The theta-logistic is unreliable for modelling most census data. *Methods Ecol Evol*, 1, 253–262.

Condamine, F. L., Rolland, J. & Morlon, H. (2013). Macroevolutionary perspectives to environmental change. *Ecol Lett*, 16, 72–85.

Cramer, B. S., Miller, K. G., Barrett, P. J. & Wright, J. D. (2011). Late Cretaceous–Neogene trends in deep ocean temperature and continental ice volume: Reconciling records of benthic foraminiferal geochemistry (δ^{18}O and Mg/Ca) with sea level history. *Journal of Geophysical Research: Oceans*, 116.

Ezard, T. H. G. & Purvis, A. (2009). paleoPhylo: free software to draw paleobiological phylogenies. *Paleobiology*, 35, 460–464.

Hassell, M. P. (1975). Density-dependence in single-species populations. *J Anim Ecol*, 44, 283–295.

Hunt, E. G. (2006). Fitting and comparing models of phyletic evolution: random walks and beyond. *Paleobiology*, 32, 578–601.

Lazarus, D., Barron, J., Renaudie, J., Diver, P. & Türke, A. (2014). Cenozoic planktonic marine diatom diversity and correlation to climate change. *PLoS ONE*, 9, e84857.

Lear, C. H., Mawbey, E. M. & Rosenthal, Y. (2010). Cenozoic benthic foraminiferal Mg/Ca and Li/Ca records: Toward unlocking temperatures and saturation states. *Paleoceanography*, 25, n/a–n/a.

Moore Jr., T. C., van Andel, T. H., Sancetta, C. & Pisias, N. (1978). Cenozoic hiatuses in pelagic sediments. *Micropaleontology*, 24, 113–138.

Norris, R. D. (1996). Symbiosis as an evolutionary innovation in the radiation of Paleocene planktic foraminifera. *Paleobiology*, 22, 461–480.

Peters, S. E., Kelly, D. C. & Fraass, A. J. (2013). Oceanographic controls on the diversity and extinction of planktonic foraminifera. *Nature*, 493, 398–401.

Pinheiro, J. C. & Bates, D. M. (2000). *Mixed effects models in S and S-PLUS*. Springer, New York.

R Core Team (2015). *R: a language and environment for statistical computing*. Vienna. Http://cran.r-project.org/.

Steeman, M. E., Hebsgaard, M. B., Fordyce, R. E., Ho, S. Y. W., Rabosky, D. L., Nielsen, R., Rahbek, C., Glenner, H., Sorensen, M. V. & Willerslev, E. (2009). Radiation of extant cetaceans driven by restructuring of the oceans. *Systematic Biology*, 58, 573–585.

Watson, G. A., ed. (1978). *The Levenberg-Marquardt algorithm: implementation and theory*, vol. 630. Springer-Verlag, Berlin.

Wood, S. (2006). *Generalized Additive Models: An Introduction with R*. Texts in Statistical Science, 1st edn. Chapman & Hall, CRC, Boca Raton, FL.

Xie, Y. (2012). *knitr: A general-purpose package for dynamic report generation in R*.

Zachos, J., Pagani, M., Sloan, L., Thomas, E. & Billups, K. (2001). Trends, rhythms, and aberrations in global climate 65 Ma to present. *Science*, 292, 686–693.

Zachos, J. C., Dickens, G. R. & Zeebe, R. E. (2008). An early Cenozoic perspective on greenhouse warming and carbon-cycle dynamics. *Nature*, 451, 279–283.
